# Supplementary material for: UK midwives’ perceptions and experiences of using Facebook to provide perinatal support: Results of an exploratory online survey
Source: PLOS Digit Health. 2023 Apr 17;2(4):e0000043. doi: 10.1371/journal.pdig.0000043 (PMC10109466; doi:10.1371/journal.pdig.0000043)
Supplement: S1 Data — (PDF) [file pdig.0000043.s001.pdf]

text

Midwives' perceptions and experiences of using Facebook Groups to support families

October 4, 2022 4:19 AM MDT

New Custom Page

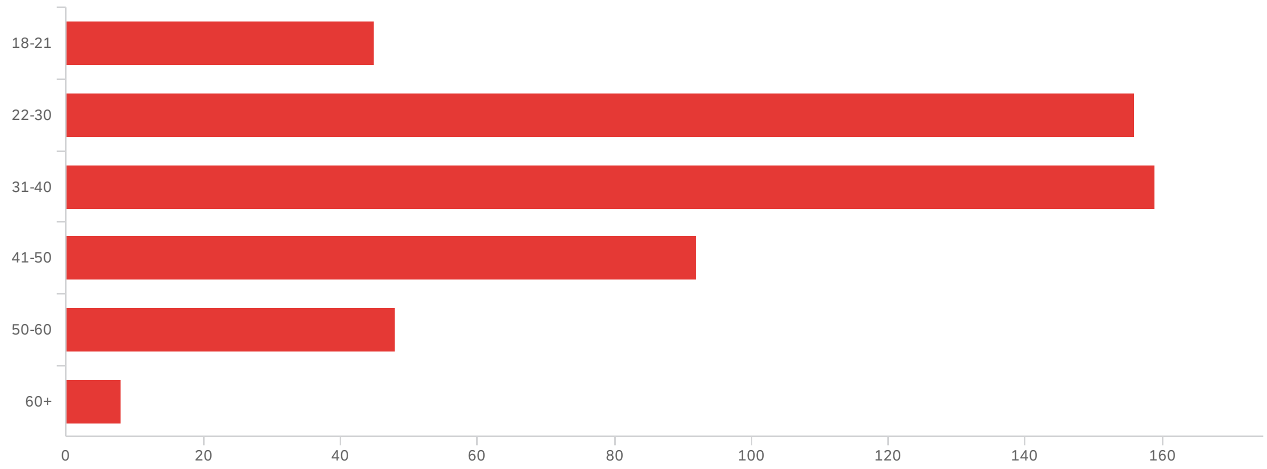

Q4 - How long have you been a midwife?

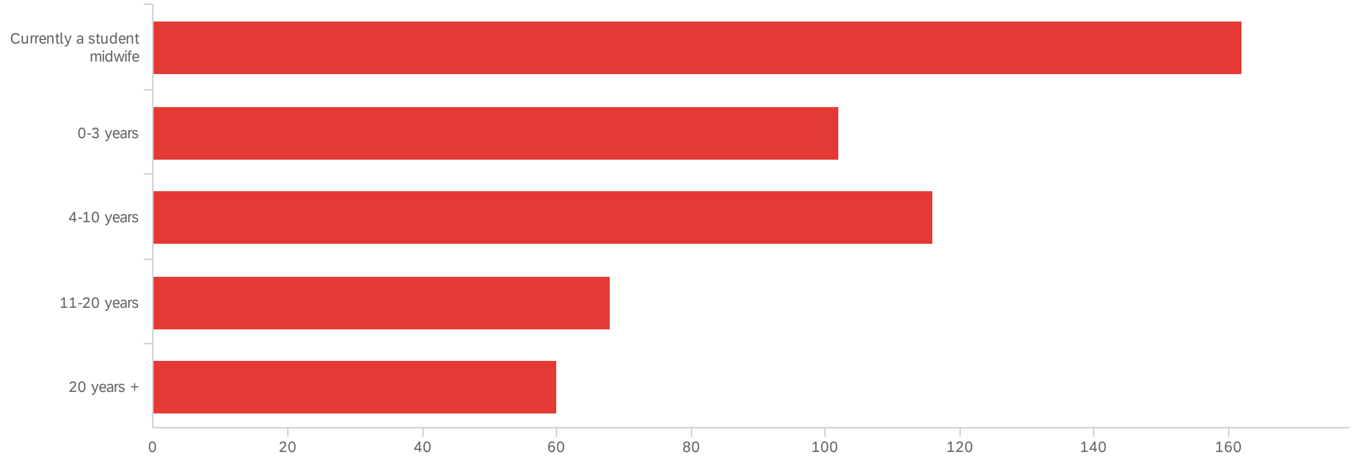

#	Field	Minimum	Maximum	Mean	Std Deviation	Variance	Count
1	How long have you been a midwife?	1.00	6.00	2.65	1.60	2.55	508

#	Field	Choice Count	
1	Currently a student midwife	31.89%	162
2	0-3 years	20.08%	102
3	4-10 years	22.83%	116
4	11-20 years	13.39%	68
6	20 years +	11.81%	60
			508

Showing rows 1 - 6 of 6

Q5 - What is your current role?

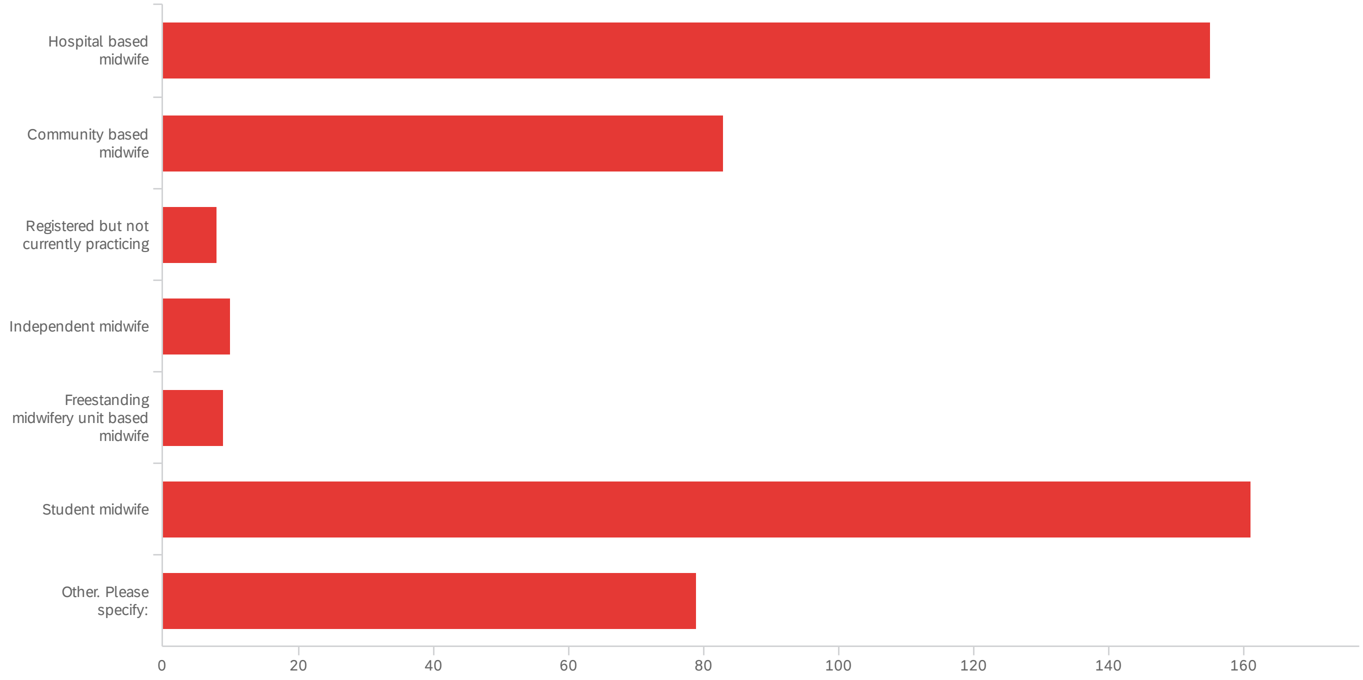

#	Field	Minimum	Maximum	Mean	Std Deviation	Variance	Count
1	What is your current role? - Selected Choice	1.00	8.00	4.35	2.96	8.75	505

#	Field	Choice Count	
1	Hospital based midwife	30.69%	155
2	Community based midwife	16.44%	83
3	Registered but not currently practicing	1.58%	8
4	Independent midwife	1.98%	10
6	Freestanding midwifery unit based midwife	1.78%	9
7	Student midwife	31.88%	161
8	Other. Please specify:	15.64%	79
			505

Showing rows 1 - 8 of 8

Q5_8_TEXT - Other. Please specify:

Other. Please specify:
Part time hospital based NHS midwife, also self employed (not intrapartum care)
integrated preceptee
Split community/hospital preceptee midwife
Lead midwife (Non-clinical)
Midwifery Lecturer
IFC
Continuity
Infant feeding
Research midwife
Maternity Matron for Community and Governance
Community midwife manager
Research midwife
Research midwife, but clinical through back shifts
Home birth midwife
Nurse midwifery consultant
Safeguarding specialist
Educator
Continuity team midwife
Regional chief midwife
Public Health
Continuity midwife
consultant midwife
continuity midwife
Team midwife, working in community and hospital, as well as freestanding, alongside MLUs and home birth

Other. Please specify:
RM working for digital company. Some clinical bank shifts direct patient care
Specialist, non clinical midwife
Continuity of care midwife
Also work on a birth centre
Jigso Midwife in Community
Midwifery Lecturer
Managerial
Maternity investigator
Midwifery manager
Midwifery lecturer
Continuity of carer midwife
Placements within freestanding midwifery led units and hospital based.
Educator
Academic
Digital midwife
Social media midwife
Project Midwife for Digital Maternity Service
Specialist midwife in women's experiences
Better births midwife
integrated/ CoC midwife
Integrated midwife
Integrated midwife (community and hospital/birth centre)
Continuity team midwife
Local Maternity system

Other. Please specify:
Awaiting registration
Midwife in a continuity team
Lecturer
lecturer
Lead midiwfe - service development
Specialist
Continuity of Carer Midwife
Bank Midwife (Community)
Continuity Midwife
Community and hospital
Community and clinical governance
Integrated midwife (working between community and labour ward)
Hospital and community based midwife
Rotational midwife
Independent midwifery educator
Lecturer/ PhD Fellowship
Private midwife and tt practitioner
Charity
Continuity team midwife
Maternity investigator
Rotational midwife (hospital & community)
Integrated midwife, CoC model carry a caseload and cover the CLU to care for women in the team
Caseloading Midwife (continuity model)
Lead Midwife for continuity

Other. Please specify:
co located birthcentre midwife
Midwifery matron
Continuity of carer midwife
Continuity midwife, community and delivery suite
Integrated community/ hospital midwife

Q6 - Do you have a specialist role?

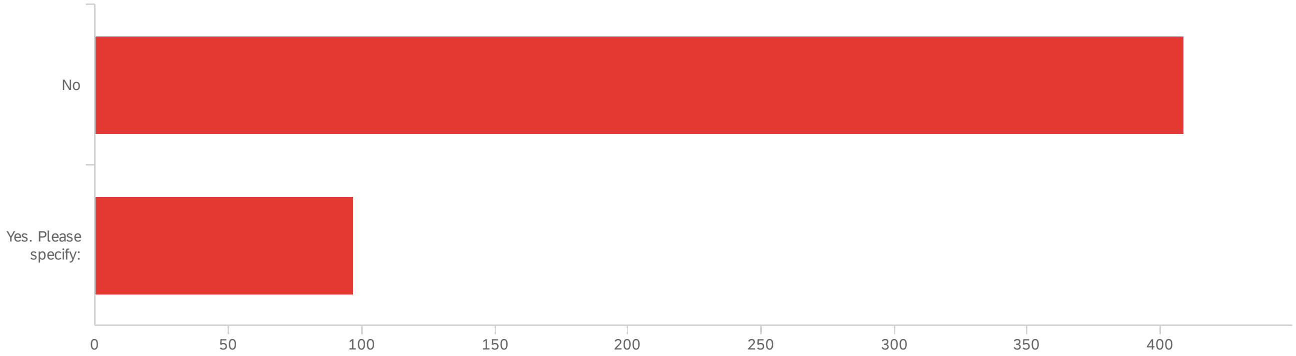

#	Field	Minimum	Maximum	Mean	Std Deviation	Variance	Count
1	Do you have a specialist role? - Selected Choice	1.00	4.00	1.58	1.18	1.39	506

#	Field	Choice Count	
1	No	80.83%	409
4	Yes. Please specify:	19.17%	97
			506

Showing rows 1 - 3 of 3

Q6_4_TEXT - Yes. Please specify:

Yes. Please specify:
Research and Midwifery Practice Facilitator
VBAC
Sonographer
Continuity of Carer Lead Midwife
Divisional Quality Lead
IT/ digital
Research midwife

Yes. Please specify:

IFC
Infant feeding specialist
Research
Breast Feeding Champion
Audit midwife
Infant Feeding Coordinator
Named Safeguarding Midwife
Research midwife
Research
Home birth
Studying for Advanced Midwife practitioner
Home birth with complexities
Safeguarding
Specialist mental health midwife
consultant midwife
Breast feeding champion
Labour suite Coordinator
Perinatal mental health and safeguarding
High Risk Lead
HDU
Fetal Wellbeing
Urodynamic nurse/ midwife
CDS Coordinator
Digital midwife

Yes. Please specify:

Digital Midwife
Digital Midwife
Practice Development Midwife
Continuity lead
Jigso Midwife
Midwifery Lecturer
Stated above
Antenatal and Newborn Screening
Homebirth Lead
Medical acupuncturist & independant midwife
Digital midwife
Research midwife and fetal medicine midwife
IBCLC
Specialist MW Infant Feeding
Infant Feeding Coordinator
Social media
Training and mentoring midwives to provide continuity of care via digital support
Diabetes Specialist Midwife
Women's Experience Midwife
Better births matron
Risk/governance
Homebirth midwife
Matron
Safety and governance

Yes. Please specify:
Specialist lead midwife community
Delivery suite coordinator
Lead MW Midwifery Led Unit
Cultural midwife
Inpatient matron
Digital
Labour ward co-ordinator/ Fetal monitoring Lead
Project midwife for Facemums
Digital Lead midiwfe
Quality lead/practice development
Clinical risk and quality standards
Continuity
Specialist mideife in early pregnancy
Diabetic Midwife
CSfM
Infant Feeding Advisor
Lecturer
IFA
infant Feeding
Deputy sister
tongue-tie practitioner and IBCLC
Specialist Midwife for Infant Feeding
Infant feeding
CSfM

Yes. Please specify:
Maternity investigator
Cpf
Lead Midwife for continuity
IT support for maternity information system
Labour ward coordinator
Jig-so Midwife
Consultant midwife
Student link
Clinical link for MVP
NLS INSTRUCTOR
Infant feeding lead
IBCLC Lactation Consultant & TT Practitioner
Feeding support

Q7 - Do you use Facebook? If so which if these apply? (Please tick all that apply)

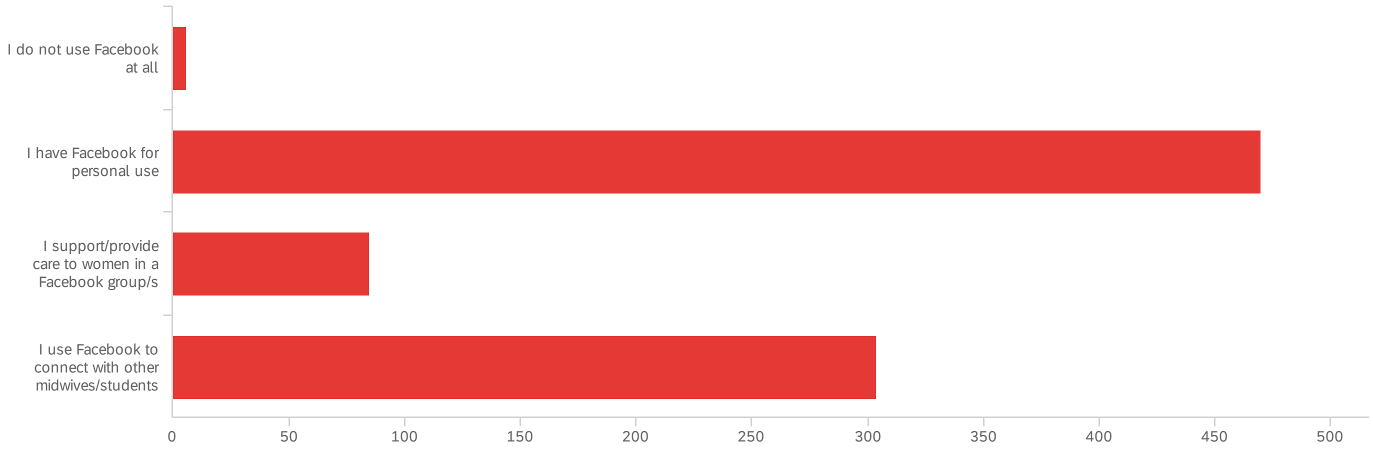

#	Field	Choice Count	
1	I do not use Facebook at all	0.69%	6
2	I have Facebook for personal use	54.34%	470
4	I support/provide care to women in a Facebook group/s	9.83%	85
16	I use Facebook to connect with other midwives/students	35.14%	304
			865

Showing rows 1 - 5 of 5

Q15 - Would you consider a role in providing midwifery support/care to women within an official Facebook group? Please explain your reasons.

Would you consider a role in providing midwifery support/care to women with...
Yes behind an anonymous or group profile, not using a personal profile.
No, the issue with midwifery support is that it should be tailored to the individual woman/birthing person and their families. Giving clinical midwifery care should always be in person because what you would suggest may differ depending on how the mother is presenting. General ‘support’ on Facebook is ok, but should never be a replacement for hands on midwifery
Maybe depending on account and security and accountability
No as I don't like social media as it isn't very social and communication over social media isn't always clear and understandable
Yes I think that in the current situation it could help women
Yes, but there needs to be clear guidelines in how the service is used I.e not for urgent clinical queries or questions. I used to use it in a previous role to promote community groups in the area and to share useful. Information with pregnant people.
No because it blurs the line between professional and personal life
as a current (just started perceptee) o would not be comfortable with such role. I thibk it could be a quick, easy and helpful tool for todays women to use and to be guided on. However, the sense of professional conduct should concider to limit medical advice and moreover the 'stress factor' in open groups such as NCT cause when problems are blown out of proportion often casuing negative interaction
Yes as I believe I could work with the trust to ensure professional on line advice
Not sure, would need clear guidelines when giving advice and triaging issues online.
Not for me
Yes, I have been involved in a breastfeeding support group on Facebook before I undertook my midwifery training and found it a useful and valuable resource
Maybe . Worried about the permanency of words being always online or screenshotted . And midwives all have different ways of working and differences in practice.
Yes, but only addition to being in practice
Yes, due to societal reasons women can feel more lonely and not everybody is fortunate to have strong female role models in their life. Although I feel FB could be a contributing factor here I also think it could be used as a support network for women if professional standards are upheld
Possibly. I am a bf peer supporter and we have a fb platform to support mums. I have seen the benefits of using fb, my concern would be that women could view personal profiles of midwives and the blurring of work/life balance for the professionals
No as I think the line between being a professional and friend would blur for the women

Would you consider a role in providing midwifery support/care to women with...

Yes, despite my reservations, my locality has a breastfeeding support group on Facebook which serves the community well and answers common questions by trained professionals.

No as prefer patient contact where body language tone and manner can be interpreted. Video calling may be possible but even then being with woman can tell you more than just what is said

Yes if done in the correct professional and official channels

No

Yes if I had the time

Yes if I personally remained anonymous. It's a lot to be held accountable to something which can so easily be misinterpreted

Yes, there are very successful official local groups for breastfeeding support (not run by midwives) and I think it could be valuable to have one for antenatal information.

Yes. With clear guidance

No, as it's difficult to treat someone without face-to-face contact

I probably would not as there are other websites/social media which I could use in order to connect with women

No, just due to being too busy outside of work.

Not at the moment. For 18 years I worked in a Trust that wouldn't allow employees to have social media groups to support each other and managers would never communicate with us that way. Now for 2 years I've been at Trusts where there are Facebook groups for the managers to communicate with staff by. It makes me uncomfortable because for me social media is for my personal life even though that sometimes includes colleagues. If go on social media out of work I don't want work issues to encroach on that and neither is it right to miss important work information that has only been communicated via social media if I've chosen to have some time off social media (this has happened). I don't disclose on my personal social media accounts that I'm a midwife and I never comment in a way that could be taken as 'professional' advice and potentially get me into bother with my employer and/or NMC. I would only consider giving advice online as part of my job if I was anonymous as a Trust employee, not as myself.

No, It's impossible to gather all the information, other non-medical professionals give their opinion diluting the impact of the health care professionals advice. It's also impossible to trace who made which comments when using a generic log in for the service and I would not use my personal account to post on line.

No, it's difficult to give advice without access to handheld notes and medical/obstetric/social history

No

Absolutely not. I've read some of the comments that service users make on Facebook and its incredibly rude. Particularly surrounding scan appointment and partners only being able to come to the first scan (covid guidance) no-one deserves to be treated like that. I think we have to ask what is the Facebook group for? Is it for people to get advice? If so how does that affect their relationship with their named midwife...why does she feel she can't trust them. Is it for support? If so I'm not sure an open forum is supportive all the time. Some people are overly negative and internet trolls exist everywhere.

Yes, i think most people these days have social media and its easy to access. Most women are on other unreliable facebook groups for information so providing one with evidence based practice for women would be beneficial

No, mixing personal life with work too much. Women should be directed to using triage telephone lines not Facebook

Would you consider a role in providing midwifery support/care to women with...

Yes

Yes. Easy and accessible for women

Absolutely, I feel social media is a powerful and convenient way to reach mothers, especially during the current pandemic. However my Uni (UWS) has highly discouraged us from using social media to discuss our career or course.

Yes! Great way for women to access support

Yes, if the trust have their own in place i would feel more supported to provide that information to women.

No

Yes. I believe its a good way to reach a large number of women, information can be saved by them abd stored for later

Yes. I think it could reach a wider demographic of users. You can also support more mothers than you can in a physical class-based support session

No i feel that infirmation can easily be misunderstood/ manipulated

Not from my personal account. Only as a 'generic midwife' profile

I would consider this. I believe that women join mum/due date/support groups via Facebook and feel that a professionally run page would ensure quality advice.

Yes, but only on a professional platform. Many women use facebook to seek advice from other mothers, which is unsafe if we can offer a professional evidence based service this is another methid of support for all mothers and families.

Yes, I have done it with Facebook project. It is an other way to support women

Yes, As part of a face to face role also.

No I would not. As a st mw, it is outside my remit. Also once qualified I would be terrified of giving poor advice and this being shared around Facebook, not only spreading incorrect advice but also as a witch hunt to myself. Potentially exposing me to online abuse.

No, i personally struggle with prociding information wothout actually speaking to the individual

In principle, however it concerns me that service users with an axe to grind can cause more harm than good - trolling the service creating concern and anxieties for other service users - we have major issue with this

Yes as not all women want to a- talk to someone b- it's quick c-if you forget to ask the midwife a question it would be used

Yes, as I feel confident in my skills and knowledge as a midwife

Yes, but only when sanctioned by y employer

Would you consider a role in providing midwifery support/care to women with...

i think this would be a great idea with certain guidelines out in place. I am a member of a mum group for babies born during lockdown (recently had baby no6) and we have helped each other through and given advice regarding maternity care. I have enjoyed using what ive learnt to help guide women and over come stigma and incorrect information, to look for research into their own individualised care but have always remained professional and referred women to their own midwives at all times. Facebook seems to be the first place women turn to for advice and whilst we can give advice its still important that they seek help from their midwfe rather than soley rely on non contact diagnosis. Its a great platform for support and information and having a midwifery led group trust specific would be a good idea, women need to know not to solely rely on this. It would be good for group classes, breastfeeding support or for community midwives to utilise too.

No - NMC reasons. Plus I believe that it will blur lines with professional boundaries and increase the risk of burnout. I strongly disagree with this idea - if a woman needs advice they should contact their community midwife accordingly

Yes, I believe it’s a quick and easy way of reaching a good number of women

If supported by professional body

No, not interested

No. Women become reliant on it and it could lead to them not accessing help in a timely manner. Women would also expect instant answers and responses for information that may not be urgent

Potentially if it was done with a professional account - not my own personal account. I’m not sure how much of the “art” of communication with women would be lost. Sometimes I think advice can seem quite blunt when written rather than said with compassion. So I think I would enjoy it but I’m not sure whether I’m skilled enough with written communication to truly meet the needs of women online

No

Yes

Yes, I’d love to! Think it would bring down a lot of the unequal power dynamics that are implicit by being a HCP

no

I think it's a useful platform but would not want to use my personal profile or for this to be involved. I think having pages where women can ask questions and have peer support are great

Yes I think it’s a good way to reach out to women and families and provide relevant information and support virtually.

No, for me I keep FB separate to work as it’s my personal social space

I would consider it as part of my current role, but not as a full-time role.

No

Yes but once i am qualified

No

In my trust the midwives who post support are the ones who run breastfeeding pages or the managers who put the practical info on ward visiting times/etc.

Yes if it was not through my personal account

Would you consider a role in providing midwifery support/care to women with...

No. I feel that it is impossible to monitor consistently and could lead to missed information, or False information being provided by non professionals

This would depend on the quality of the group

No, as we have dedicated staff to do just that

No. The intellectual property is owned by facebook. The security of facebook logons are.frequently compromised.

Yes as long as the trust approved

Yes

No, I think it risks the loss of personal care

Probably, but would rather have a professional account to do so instead of a personal account.

Yes, if I was allocated time to do so. Although I'd be cautious with what advice I'm giving in order to protect myself.

Yes. I enjoy caring dor women and this is a modern day way to do so and ensure people are receiving information through all options

Yes because women turn to Facebook for support and advice, it would be good to know they are receiving the right information.

I don't think i would. My concerns would be if we are getting the whole story from the woman, it's difficult to assess people when you're not face to face with them.

Breadth of potential use

Possibly, it seems very helpful for women to have a place to ask questions and get a quick and factual response, but there are also risks to offering information online in that you won't know the full story of that women and her baby.

yes, as long as the information is evidence based and not personal opinions and it is not judgemental either.

No, the availability to share posts and information given that may be woman/area/trust specific could mean that others apply it to their own scenarios and therefore pose a potential risk in that individual information has not been sought, taking all individual factors in to account. Where the culpability and accountability lies is dubious in my opinion

yes, women & families are keen to use social media. Its a good platform to reach many women at a time that's convenient to them. Good for reminders & information sharing

yes, on a professional group. There is a demand for the support.

No, not confident in my advice

No, it is easy to see when another midwife covers my clinic that sometimes the information we provide is different

no, I think this crosses a line from professional to personal and i would not like the idea of women being able to find out my information that easily.

No as Facebook is a public domain and I feel care is confidential and private. Advice given my suit one but not another. Women may twist what is said. Don't mind using Facebook as a platform from basic things like SIDS / RFM info. Nothing individualised

Would you consider a role in providing midwifery support/care to women with...

No, don’t feel it is very confidential and interaction may not be secure, feel telephone calls and face to face conversations are a better form of communication

Yes. I feel this is anathema way for women to seek non urgent guidance and reassurance.

No

No. The risks for professional misconduct are high - I have witnessed many midwives berated on Facebook following interactions I know to be impeccable. Our trust doesn't have official support but there is one affiliated with our trust, run by volunteers and CCG professionals. Despite input from midwives the conversation amongst women invariably becomes dangerous in its recommendations and there is no way to implement baby friendly standards. Individualised care which has been provided becomes a source of discord, such as one occasion where another woman's care was discussed publicly - the lady had been returned to the obstetric unit due to PPH and sepsis. Another woman had witnessed what she felt to be the woman receiving preferential care (her own room during covid, but of course this was because she was unwell and needed close obs). Without her knowledge, this poor woman was discussed at length and bullied. I reported it to admin and it was not removed. I commented and intervened and it still was not removed. This is not an unusual occurrence.

Yes, as long as it stays a private group

Yes. I’ve seen the impact that the Facemums programme has had in my local area and I feel that women would benefit from professional, moderated information from midwivea

Yes. Necessary though to include people that could be excluded from this way of supporting

aid co.munication and information giving

Yes, fast and convenient way to give evidence based care to women. Easy for women to access and share. Eay to see while acrolling on thwir facebook

Yes. As an additional resource that is accessible to all.

Maybe if paid enough. I’d be reluctant to as I think it’d be really hard to switch off from

I would in an official capacity if it was part of my role. I dont feel it is appropriate to do so unless part of your role, as you do not know the woman's full history or circumstances

In a closed group with specific rules. I have actively stalked by a woman on facebook and messenger can be very scary

its a good medium for information, social media widely accessible to women and convenient for them.

Yes - if used appropriately can provide valuable information and support for many women, but particularly for those who speak English as a second language.

No I don’t think it is safe to give advice without the full facts

I woukd want it to be totally seperate from my personal profile etc.

No

I would comsider it in the future however it doesnt seem very confidential, others could resd the post, midwives could become too emotionally involved. Also personal profiles could be on view.

Would you consider a role in providing midwifery support/care to women with...

Yes - but would have to fit in with work/life balance

Yes i feel that there could possibly be a role in midiwfiery support to women as it is quick and easy to access and relay information. However I do not fee that we can care for women in this way as we are not able to get an accurate assessment of the woman.

I would consider this, however I feel I would need more training on how to respond to comments/questions that may be quite negative

some women have social anxiety and feel more comfortable in asking questions and raising issues online without a face to face or telephone meeting

No, I feel women should have formal interaction with midwives they have built a good relationship with to inform their care

Yes I would consider a role in supporting women via social media as I feel it makes support accessible for all women and is a really useful tool in empowering women to support eachother

I would like to do this, but have always been told not to use social media to give any kind of midwifery advice.

No, don't like to post on social media

Social Media is a vital and huge form of communication we should be using.

We are in an age of social media. People expext to be able to use these methods to communicate. As service providers we need to be able to adapt and use the same platforms as our service users to ensure we provide evidence based and accessable care

no i find it inpersonal

Not sure, I would want to do more research about this. I think FB is great for sharing information and it would be great to myth bust. However, people often misinterpret the things we say and I'd worry about this being more so on social media.

No

Not sure as women may contact you at all hours when you're not working and want to put midwifery to one side

Yes to ensure advice is correct and safe

I consider the majority of our women live their lives using technology. Midwives need to embrace this means of communication to enhance maternity care

Yes, being able to support mothers is my job and if that includes offering support via facebook then I'd be happy to do so.

No, Facebook is not the place for midwifery care. You are unable to view the social aspects of the woman's life in a holistic way through a computer screen.

Yes, if it was not provided via my own personal facebook. I find it difficult when lines are blurred between personal/professional life.

No, monitoring the page takes up a great deal of time

Yes. I think the use of social media could be helpful for posting evidence based information and directing women to local support. And would be beneficial if caseloading

Would you consider a role in providing midwifery support/care to women with...

I am on some forums, the advice taken on by others on thread is quite scary, If i come across mis information i piont them in the right direction

Yes. I accessed peer information and gave some over the years. It would be good to have trained information on hand

A lot of people use social media for hours each day. It is a quick and easy, although does need to be used with care.

No

Maybe but I think this would require special training as to what should/shouldn't be said etc

In the future yes, i think a lot of women use social media & often use it to get information. Its important to provide the correct information, & i think a group like this would be really beneficial. Especially to women who perhaps feel uncomfortable talking to a midwife about something in person.

Yes. Facebook is a part of many mothers lives and therefore they may be more likely to access support and information by a route that they are familiar with

No. Not sure I would like to as I decline to be abused via social media and have had this once or twice when commenting on issues

Yes. For those who may have difficulty accessing care in hospital based situations or them not havubg the confidence to ask or talk through their worries face-to-face, i'd feel this may benefit those women who need. However, I am aware of the trust in which my placements take place, often have work mobile phones that the women can contact a midwife on. I also think it'll be a positive for boosting empowerment among other professionals, women themselves and creating a safe and supportive community. However, I would be concerned with the policing of comments/replies to questions asked by women. And appropriate content.

Unsure. I would be mindful that my philosophy of practice does not marry with the expectations of some employers. I'd also be concerned about being judged by others who do not share similar approaches to mw care. I would feel I'd have to "tow the line" in that I could only offer support and advice that is aligned with institutional midwifery care.

I would. I think there needs to be a range of flexible options. FB shouldn't be an alternative to other methods of getting info but women are seeking information on social media and for midwives not to have an official voice there means misinformation is more likely.

If there was clear guidance for this yes.

No

We need to support where women are/what they use

Yes because face book is so accessible to patients

Yes to provide support in times of need as some women reach out for help in this way. Having qualified professionals providinf that support will help stop the spread of misinformation and provide evidenced based care.

Yes, as long as it was officially sanctioned. I think it would be a great source of support and advice if used correctly

I would however I feel there would be blurred lines between work/home life. If the support was limited to certain hours then I feel it would be good to use

yes

Not confident with how social media works - not my strength at all.

Would you consider a role in providing midwifery support/care to women with...

Yes I think it's a reliable way to get information across to women who are otherwise anxious/nervous to reach out

YES - AS I FEEL IT WOULD ENHANCE THE CARE WE PROVIDE AND MAKE MOTHERS FEEL CONFIDENT IN CONTACTING US AT ANYTIME THROUGH THE USE OF SOCIAL MEDIA

I have been told by my university that we must never interact with mothers on social media, I imagine they would support a professional approach but as they have given us no training and been very negative about social media I wouldn't dare (and my background is in digital marketing).

Yes

Yes i believe it would be extremely beneficial for women to be able to access help and advice 24/7, also may help in providing a continuity of carer too.

Yes- it's an official Facebook group

Yes, within an official group although would feel uncomfortable using my personal profile. I would like more training on how to provide support whilst remaining professional.

No

yes, i think we can reach out to a wide audience of women with correct and useful information

yes as this is clearly where women are accessing information so we as professionals need to move with the times!

I would not. Women prefer face to face support and reliance on Facebook enables nhs trusts to avoid that responsibility

No

Yes as it's good for experience

Yes, in a closed group

Yes but only if this group was not using my personal Facebook. They're needs to be a boundary a

Yes, however without an official group and guidelines in advice that could be given i would be very worried about getting in trouble for saying something unapproved by the trust

Potentially however i feel there are circumstances where it would create more problems than solve. I think general information is good to share via social media but patient specific should be done face to fave or via phone wherever possible

Yes, we need to be accessible and sometimes women would rather text or message than call

Yes as I believe Facebook / social media is an accessible platform for information, as long as it is kept confidential, safe and professional. It may be easier and quicker platform to gain information.

Yes. I currently run our Facebook page but I would like to run a Facebook group to support the women on my caseload.

Yes if supported by my employer.

Would you consider a role in providing midwifery support/care to women with...

Yes, it's a relevant platform in 2020. Some use it because they are unable to socialise or have no 'real' friends so can be a source of good care for lonely women

No. Facebook groups are open to bullying, misinformation, potential issues with data protection...the list goes on!

Social media is current and we need to adapt to the needs of the service users. I am a community based midwife caring for quite a range of women in ages/social groups/ethnicities. I've had to adapt how I communicate to the women- for example my younger girls will not call or ring for advise to the hotline but prefer to ask for my advise. These ladies also would not remember to come to appointments if I hadn't of text them with it in the first point.

Yes, ensures that midwifery care keeps up with society and allows women from all backgrounds to engage with it. I think it may allow women to feel more connected with maternity services and other pregnant women,. A Facebook group should not however replace other means of contacting/engaging with maternity services but help to add to it.

I would as long as there was clear guidance and a robust pathway so that information is kept up to date.

Yes as you can reach a wide audience

Yes! Lots of woman no matter their age use Facebook daily and so it would be a great opportunity to reach out to more women and give daily support.

Yes, as it is now the new norm for people to use social media as a way of connecting and via platforms like facebook women may be more inclined to seek support should they needed. Especially now, during the current pandemic.

I would if it was relatively anonymous. I wouldn't want mother's finding my personal page - not that there's anything to hide. I'd just be wary

Yes. I am aware of other local trusts offering support on social media and see that it works well. I am aware that younger women may use social media as the first port of call for advice and it is important that this is evidence based.

yes. as long as there were strict boundaries in place (e.g. only using it during work hours, clear expectations about what kind of support can and cannot be given)

Yes it would be as other midwives can show support and mothers can also find support from other women and midwives. At a clinic appointment it is only 15minutes and women may not have the time to ask all the questions. Furthermore, a lot of women use social media and find it helpful. Women are not interested in reading vast amounts of leaflets but rather get an answer when asked a question

Yes if done properly and via work computer account etc. Should not be accessed by those off duty or linked to staff personal account.

No, facebook is a social media site and not professional

Maybe, but I'm not that tech/social media savvy, not that interested in social media to be very good at it

Yes. I see it as the same as answering queries over the phone.

Yes, we need to move with the times. Lots of women are more likely to interact with services if they are available in a format that is easy for them to access. I suspect that social media support will make services far more accessible to those who find it difficult to travel or for those who find leaflets/written materials difficult to understand.

Yes as it is a platform that women can access easily and I would be happy to provide evidence based information in this platform

Would you consider a role in providing midwifery support/care to women with...

Possibly. In a trust based capicity it can be good to provide women with trust soecific information and helpful general information e.g. infographics on normal foetal movement, trust based leaflets. I think generally informing/supporting women in an open forum is problematic; it can provide conflicting information that is either based in a midwife's perosnal opinion or may be trust specific and therefore not applicable to the woman or giving her false information/expectations (e.g. giving advice RE what can happen with homebirth or BBA could differ wildly between somewhere with a low birth rate in the countryside and somewhere highly populated such as inner London)

Possibly- depends on the type of information

yes if it was a role supported by my employer with clear guidelines

Possibly, though part of me still feels more comfortable discussing things over the phone or face to face as I would feel more confident tgat i could ask about and/or access all relevant history and information better that way.

No. I have seen women posting for advice, when they should have contacted their midwife.

I have when sick been used to man a Facebook ‘ask the midwife’ page to answer questions re covid. And ,any general midwifey questions. This releasing ward phone ones enabling labouring women to get through quickly. It’s a fab service that I’ve been happy to help with.

I would consider running the trust Facebook page giving updates on the unit and equipment we have. I wouldn't give out any pregnancy related advice as I wouldn't know a history and this is better suited to the community midwife or obstetric team/consultant

Yes, I feel it helps women especially in this day and age.

No, I do not feel it is a good platform as it may be confusing and women may turn to it when they should be making immediate contact

No. I’d rather face to face interaction

At this stage of my training no

Yes. I feel this would be a valuable source of support as so many women fall to face to find support for their needs often getting unsolicited advice from non professionals. It would be good for them to go somewhere to have professional advice.

Yes, in the age of social media most women accessing maternity services are using it. It may also remove some of the barriers that may prevent women from accessing advice from other avenues, such as feeling like they are “bothering” professionals and feeling intimidated or condescended by professionals. Social media is a more relaxed avenue to access information.

Yes, I feel women would benefit from this support especially during COVID

No, because I don't think I could give good care for a lot of things online. Unless it was similar to triage phonecalls, and advising when to come in to be seen.

Yes, would like to participate in new steps forward as care progresses in to the modern times

Yes once qualified however no as a student midwife- concerned about providing false information on such a public platform.

Yes, to help women with any concerns they may have during pregnancy and afterwards

Yes- women often turn to others for advice prior to contacting a HCP and i have seen other mums give out unsafe advice, for example telling someone with reduced FM to invest in a home doppler

No. Everything you say can be knit picked and is there in written format. It is also hard to give personal advice on a public group

Would you consider a role in providing midwifery support/care to women with...

I support the idea of it but feel it doesn't fit well with my skillset. Also as a male, I find it harder to discern (and be discerned) the line between providing advice as a HCP and mansplaining. Much easier to do in person to form a professional trusting relationship.

Yes if specific guidance was provided

I would consider it on the proviso that I would not use my personal Facebook profile

Yes, I think it would encourage women to be more open and allow us to build better relationships

No

Yes- can be useful alongside face to face care.

I am one of a select few that monitors our Trust facebook page answering questions and providing information.

I would as long as there were clear parameters and safety nets.

Yes if I had a professional fb profile

yes I do, as long as this is a professional account and not from my personal one

No because I think most of information is put out there and women cannot ask questions about it which means that when confusion arises then misinformation is shared between women. For example, there was a post put on about FSE leads and it lead to a number of women sharing facebook posts about how midwives 'cut into the baby's head' and that women should refuse them if asked.

Because these days FB is main place of gatherings and most pregnant ladies use FB to get information about pregnancy,Labour and postnatal care

Maybe, I think it could help some women, but blurrs professional boundaries and worry that women will be more 'unfiltered' in what they say online.

Yes

No, it is easy for things to get twisted on social media

Yes if it was supported by the trust and only health professionals able to provide ebp

I don't think Facebook is an appropriate platform as information relevant to advice given may be missed.

Yes, if it via proper channels, and is monirored and safeguards in place

Yes! The future of communication is online. Women are turning to online sources to find advise and information fit their problems. Therefore, it is highly useful to have qualified professionals online to provide evidence based information, support, direction and education.

Yes, only within an official FB group where comments could be moderated

Yes i would because i feel social media is under utilised with midwifery

No, I feel that using Facebook brings work into a platform I usually use in my private life. Also with so much misinformation being given we shouldn't direct women to Facebook where they can't see the true person they are receiving advice from.

Would you consider a role in providing midwifery support/care to women with...

No, everyone if different. We do not have their notes and a true background. They should serk help from those looking after them

Yes

No, i find it too personal

Yes, an easy way to interact with lots of women who all require similar info or who dont want to ask their own mw.

Absolutely not. Would be worried about confidentiality, being hacked, being stalked

No. It is totally unprofessional and unsafe for both mothers and me as a professional.

Yes because it's more relevant to the current childbearing generation

Yes, social media has its role in providing care. My only reservation is if women after questions that may result in a negative impact upon their health or their babies healthy eg. Fetal movements or bleeding. And of I they at 2 am in the morning and you do not reply till working hours 9-5 and then there's a negative outcome I could find that difficult. And then o would find it difficult to switch off when not in working hours as I'd feel responsible for answering 24/7

Easy access for mothers, especially younger mothers. Quick to use. May be beneficial for those with communication anxiety who struggle to phone their midwife to ask questions

No not really, I've discovered being pregnant myself using pregnancy forums mothers are using social media to undermine and at best contaminate the good name of midwives.

Yes

Yes providing it has correct boundaries and guidelines. I feel that many FB pages are there as a sounding board for women's frustrations and, whilst their voices need to be heard, this can fuel fear and anger (especially when out of context)

Yes. In a previous role this occured alot and frequently. Clinical concern were dealt with swiftly and in person over the telephine or face to face due to having continuity. General advice was fantastic but only because all midwives were allowed to comment and all midwives had the same ethos. This cannot be achived in the NHS instead it is 2 midwives who have access which highly restricts the information putbout as alternative therapies or theories are not allowed to be voiced. The NHS run facebok groups are dull, stick to rigid posts. There is no inspiration, compassion or alternative views to their own.

I would, I think it would be a useful way to reach women instantly at low cost, but would require training and support from the trust

Yes. Beneficial in particular to women who need support an unsociable times.

No - prefer face to face care

Yes but only to enhance care, not to replace face to face care

yes, i feel we need to move more to opening up social media to our expectant mums. Especially the younger women, they are often much happier speaking and asking more remotely.

Yes. The highlands is a wide geographic area with women up to 3hrs30mins from the teritary unit where the majority give birth. To have a link would redice anxiety

If it was established by the trust and regulated

Would you consider a role in providing midwifery support/care to women with...

Yes because I feel this is a way for women to answer questions with having a time limit.

Yes, as we have a specific criteria as to what is posted on there. It is generalised information not replies to individual questions

Yes because it may encourage women to seek help who wouldn't normally

Yes i think to many women rely on bad advise from others

Yes with adequeate training and support on the trusts social media policy

possibly, i think it could work

No, unfortunately through the pandemic it has given women and their families a platform to criticise Health Boards, Midwives etc to the point where some of my colleagues have been personally named

Yes I would. Anenatal education provisions need to be improved and this is an accessible way to do this

Yes, if it was set up that it was safe for everyone to post information about themselves on.

Yes, if official training and guidance is provide

yes, as long as the midwife is knowledgable in all areas, especially feeding issues

No as i believe there are other more professional platforms that this is suitable for

Yes as many mothers may not be able to leave the house for certain reasons

Yes, i think this is more so needed in the current pandemic. Ive need my local Trust communicate with women effectively using facebook. With antenatal classes i thought that.it worked well, alot of information is given out and with it being online mothers and partners can go back to look at them if need be

In terms of sharing experiences I think it's nice to hear positive stories and feedback however my concern is giving health advice, other women could become anxious with what they read also if questions are not answered quickly enough could it leave to adverse outcomes.

no

Yes if it was on a group Facebook page as opposed to a personal Facebook

Yes, it's a good way for lots of women to access information as share their experiences

Yes

Currently, no. I don't feel confident enough as a student midwife to provide this as of yet.

I'd be very unsure without more information! I'd feel worried that women may play down their symptoms or exaggerate them, or simply not know what's happening to their bodies, so if feel unable to confidently give advice. It is also a risk that other people can comment on posts, and there may be lots of conflicting results leaving women more confused than they originally were

If it was a way of answering general queries that would have no major impact on their health outcomes. But not to use it under my own name.

Would you consider a role in providing midwifery support/care to women with...

Yes when qualified. I believe that more mothers are likely to access care and support on social media

If it was supported and organised by the healthboard and monitored to ensure that information and advice was given correctly and it was safe then yes I would be happy to support and sublime to women’s care in this medium.

No - I know Midwives who have got into trouble from the Trust for advising women

No as it would be classed as official documentation possibly undertaken without knowing a women's history/no access to notes. Also I do not think data is secure/confidentiality could be breached

Easily accessed, familiar platform for many women

I do not wish to have to use social media to be able to care for women

No. Social media can become dangerous. Lack of evidence based information can be shared as comments. Disagreeing with information can become conflict putting midwives at risk of complaints against them

Accessible to women and their families, majority of the population use facebook

Yes but only when face to face is unavailable, for example with covid or underfunding

Possibly, but I would need to have dedicated time, otherwise I wouldn’t time off from the job.

I personally would not. There is a way that you can read things on line that may come across in a different way as to how you’d say it in person. I would like to say I’m a lot more confident giving advice in person where we can have a face to face conversations and read each other’s cues

No, I prefer to care for women face to face/when I am in the work setting

I think an online midwifery support group could be a good idea, i would worry about blurring the lines between work and free time for myself, personally, i would probably feel obligated to reply to messages etc when not officially working. Also i dont believe facebook provides adequate confidentiality for any medical or personal information as a platform for such official groups.

No - online conversations can be taken out of context and are often misinterpreted unlike a face to face or telephone conversation

No because I want to keep my social media for personal use

No as I am already very busy

Yes

Possibly, depending on the size of the group and it’s location

Yes, it may be a lifeline to some women if done correctly.

Yes

Yes I would, if it was set up differently. My personal Facebook is for personal family and friends with picture of my family and days out not midwifery related. I wouldn’t mind setting up a separate account to support women but wouldn’t like them on my personal Facebook as that is my time to switch off from midwifery. But happy to comment in groups ect for advice but not having them directly as a friend.

Would you consider a role in providing midwifery support/care to women with...

No, i think you have to be careful. How information is shared on social media. Sometimes women will share and believe others advise and ecperiences over a midwife. It can be impersonal too

no as I feel I cannot support women on social media the way they really need as I do not have the full history of what is happening

Yes. It’s a commonly used platform and I feel the support would reach a large number of women

Yes. It is a powerful vehicle to reach a wider audience.

No, it can easily become a slanging match, I wouldn't want to jeopardize my registration.

yes as some women may prefer support over facebool

Yes, I believe contemporary society is hugely influenced by social media. Social media provides an accessible source of support for women and their families and I feel midwives and students should use this to their advantage.

Yes, I think it’s a good way to also share information with women and have them make friends too

Not my area of interest or expertise

Yes, I feel that during lockdown, the trust has provided a lot of useful information online for women and their families, this could be done routinely

Yes if given clear advice of how to use safely and effectively

No as you may not know a woman’s full medical history

Yes

Yes but not through my personal account. As much I love caring for women and passionate about midwifery however, I have a personal life that I like to keep separate. I’ve seen a lot of community based midwives give out phone number and it having negative effects such as women getting angry as they not getting immediate replies or not contacting triage because they contacted their community midwife and as a result don’t think women having access to messaging midwives through Facebook for advice is a good thing and they should take the appropriate channels to do so.

No, I feel women need human contact

Everything is going online now, we need to find ways to adapt patient care to give them the best care and imformation. Not all information on the internet is true so it is important to lead women to the correct sources of data and information

Depends how official it is. It’s always best to provide support physically, so you know for sure what is going on and you are providing the correct care. I would be afraid not seeing a mother or baby physically that something might be missed, or blown out of proportion on the mother’s side yet that is expected If you understand. I.e pain/severe pain

Yes. I have fears that this would not be well supported within the negative culture i currently work in.

Yes, I’m confident I can be professional in this setting and feel it will reach more women and their partners/support group.

Yes - With the correct guidance & Support

Yes I would in the future. I feel that it would be a good additional way to provide support whilst they are in the comfort of their own home, some may prefer to ask questions in a facebook group compared to face to face.

Would you consider a role in providing midwifery support/care to women with...

Yes possibly, I feel it would be used well by women. However I would have concerns that information or comments could be taken out of context.

Yes, social media can be a great way to reach and connect with people that otherwise would not engage fully in services- such as people it attending antenatal classes etc

No, I think this would be a very dangerous step into using social media. Each woman and pregnancy is different, offering individual advice is really important to healthy outcomes

Yes, however limitations would be needed.

Yes I think it could be beneficial for women

I would, but as a group admin for example. Would rather not from my personal account as could cross boundaries.

yes providing it remained professional

I would only consider doing it if employed to do it specifically, with very clear rules and guidance.

Yes I would so long as it was a platform I could use away from my personal page and there was a comprehensive policy put in place.

yes if approved by the trust

Yes,

No

Yes because the women will get professional advice but mothers and other people who have experience can also add their input

Yes. I believe it may be easier to reach some women via social media who may be reluctant to accept support otherwise.

Yes. Sounds interesting

Potentially but not on a personal account

Yes as especially at the moment during the pandemic the support we can offer women has been significantly reduced.

Yes as it is easy accessible to women of all cultural, social, and ethical backgrounds.

No, I do not think it is a suitable platform for providing midwifery support

Yes I believe the ease of access helps to improve care given and can facilitate good communication and advice sharing in a quick format

Possibly

Yes. I think moving forward, more women are using social media to access support in pregnancy from professions and peers. It would benefit women if a midwife was providing online support and information as they would be receiving more evidence based info rather than hear-say or other people perceived experiences.

I think it's a great way to support women, however I'd feel quite vulnerable and potentially open to criticism

Would you consider a role in providing midwifery support/care to women with...

No

Yes, I feel that this could be really beneficial in answering questions, supporting women and relieving pressure on triage areas if women were provided with support which could also help with Saving Babies Lives

absolutely. As a community midwife i see huge gaps in antenaral and postnatal education and support and the only groups that seem to use facebook for this purpose is nct and similar paid antenatal groups.

Yes. If it was fully monitored and only the midwife /midwives giving advice I think it could be a really valuable resource. Especially for younger mothers

Yes. Many women may feel more comfortable in reaching out for support via Facebook and may do this in the first instance before verbally informing their named midwife. A Facebook group could be helpful to mothers who feel alone, to socialise with others and form friendships. It is a quick and convenient way of communicating and providing reassurance or support to pregnant women, as well as ensuring they are aware not to rely on the Facebook group for emergency situations.

Yes. If there were good IG processes in place and guidelines then I would be happy to

Unsure

not inpresent covid times. Wehave a family assist group.

Yes

I would worry that is becomes a sounding / midwife beating board. During covid the ability to berate and shame trusts and midwifes has been active by certain types of personalities. The managment of the site would have to be car fully managed and can almost be a full time job.

Yes if it was an assigned role from my trust and not from my personal account.

I would, but not from my personal page. Would use Trust page or a specifically created professional profile

Yes. Social media is so widely used these days for various aspects. I feel that with the ongoing struggles of childcare, work schedules and other life factors, accessing support from healthcare professionals online would be an ideal way for many pregnant women.

No, to hard to manage and make sure that people in the group are who they say they are and trustworthy

Yes I think it reaches out to a lot of women who are reluctant to phone their midwives for advice for one reason or another

Yes if it was agreed and supported by the Trust. Currently restrictions on social media use would mean contact with women on FB would be seen as inappropriate.

Yes if clear boundaries were set

I would not as I am only a student midwife and I do not believe I should be giving any information other than for them to contact their midwife if they have any concerns. My advise could be wrong and I would not want to put any woman/baby at risk.

no

Yes i think many women would actively engage with this type of support.

Would you consider a role in providing midwifery support/care to women with...

Possibly depending on time constraints etc

Yes. It’s a good way to communicate with a mass group of women at once

Yes I would - many pregnant women use FB & during covid with less routine face to face appointments FB contact could be reassuring.

Only if there were strict guidelines to ensure women were being provided with reliable information. And guidelines to protect my role as a midwife. I would be worried about people misusing the information or passing on information to people whom it was not tailored for

not yet, I don't feel I have the confidence/experience as a midwife yet

No,

I think providing information in such a way is too permanent in a career/lifestyle in which guidelines and advice varies and changes so much. Unfortunately, social media can be a monster and if an error occurred in the wording of/provision of advice then there is no definitive answer to who has gained access to incorrect, and potentially harmful information.

Absolutely, I feel that supporting women both in person and online makes a huge difference and the convenience of facebook is evident. Research I've read points shows midwives are fearful of retribution when engaging on social media. As a student midwife I am part of several groups for breastfeeding peer support from when I was a breatfeedjng mother and I would love to feel confident in providing support to women but feel it is out of my scope as a student in an online capacity. However if it were advocated trust wise I would be more that happy to support.

Yes - we should meet the women where they are

Yes. Social media is so widely used now, we need to start recognising it as a way to get women to engage & reach people that would not attend groups or classes.

Yes although not exclusively. I feel that social media gives women the confidence to seek support in areas they may feel self concious or embarrassed about expressing face to face.

No

Yes. It can support harder to reach clients and provide peer support

I think it’s extremely important to be able to keep personal life and professional life separate and although I think some woman would benefit from this, I do believe that some could become too fixated on a support system/contact with midwives! I think maybe a website with common questions and videos providing information would be much more effective for both midwives and mothers/parents! I think alot of midwives feel stressed and quite run down with work and short staffing as it is therefore I think a role like this would add to this stress and midwives might struggle to switch off which in my opinion is SO important in maternity care!

No

No. Without having access to a woman’s notes i would not feel comfortable providing care over facebook. However i would be happy to signpost women to relevant agencies which i feel may be of benefit.

Yes. You could send links. It’s a way women feel comfortable but would have to be manned 24/7

Supporting mother's is one element of our role as midwives

No. Facebook for me is social and not educational

Would you consider a role in providing midwifery support/care to women with...

yes

Q17 - Do you think additional post midwifery registration breastfeeding training/experience is needed to provide breastfeeding support online?

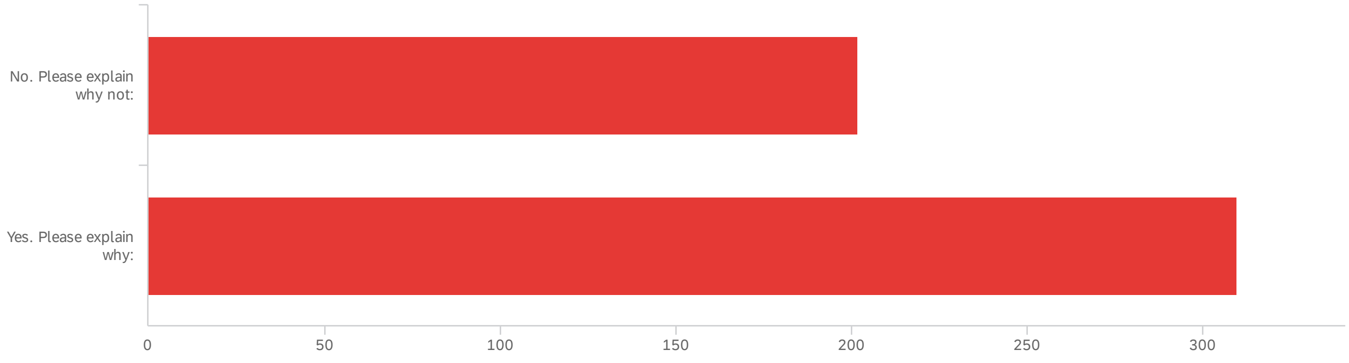

#	Field	Choice Count	
1	No. Please explain why not:	39.45%	202
2	Yes. Please explain why:	60.55%	310
			512

Showing rows 1 - 3 of 3

Q17_1_TEXT - No. Please explain why not:

No. Please explain why not:
Training should improve practice but it can be just a means of gain ing training /CPD
Not if you are experienced and updated
We have a lot of exposure in supporting women in the community, we also have a fabulous infant feeding team to support midwives as well as mothers
There is a significant emphasis on bf on the degree programmes therfore nqm's should be well quiped to do this already
It is easy to explain
I feel support is better provided in person than online
Training is constantly updated so extra qualifications not a necessity
We've basically been doing this over the phone for a long time.
University was BFI accredited and provided good education

No. Please explain why not:

I believe what we will learn on the course will be enough to provide support on any platform.

Not sure that midwifery support is valuable mother to mother trained peer support I feel is , with supervision

We do it face to face daily

There is enough opportunities in the curriculum for midwifery. There are adequate standards to meet to qualify

Mandatory updates on breastfeeding yearly within our training.

Breastfeeding support is part of the Midwofery curriculam

Have had ample training and participation in university and in placement

you have thorough training throughout your degree an din practice regarding BF and I don't feel further training on it is needed. There are a lot of women needing support in this area and with BFI etc there is always resources to go to.

Peer supporters provide excellent help online

All midwives should have the knowledge

The role of a qualified midwife already requires knowledge of breastfeeding

reinforcement of basic messages is often enough to provide first line support

At least not with my university. We have been afforded extensive and independent learning surrounding breastfeeding and anatomy through my 3 years at university and been supported during placement tonwork alongside breastfeeding peers. I am confident that I would be able to support women without this additional learning, although if it was offered it would be something I would definitely take part in.

Our university course includes breastfeeding teaching in each of the three years

My clinical role supports my online role

All midwives should have a level of expertise to provide support however extra training is always beneficial

Experience is gained while supporting moms

It’s the same whether the support is online or face to face

Breastfeeding is breastfeeding whether through a computer or face to face. I think younger people know about the dangers etc of social media use, and as the argument at the minute sharing a bikini pic doesn’t make you unprofessional. I think we also need to realise midwives are just normal people too. As to say, all the additional training I’ve seen around social media, really seems to focus on dos and donts, and scaremongering that you must always present this ‘professional’ act, I think it hinders

Depends on the Facebook page role and what advice is given

BFI trained at trust

should be the same face to face or online

No. Please explain why not:

We are all trained to a good standard and experience counts as much as further training

we have continued training in our role

Not if you’re a qualified midwife. Although this support online is difficult as a lot of it relies on observation.

Advice given online is always hard to give. I think advice should be tailored but often mothers should seek face to face help with a professional

Our undergrad students do lots of work around BF and our Trust has mandatory BF updates with an annual assessment by the infant feeding team

I believe the knowledge for breastfeeding guidance is included within the degree and experience as a midwife however, midwives should recognise if they lack knowledge and seek further training.

Breastfeeding support is part of the midwives role

Part of midwife role

Peer supporters/other mothers can give valuable advice

However would be amazingly beneficial. So many midwifes don't always stick to the facts

the training and hands on experience is more than adequate.

Practical experience is best

We have in house mandatory learning which is on going through out my career which keeps my skills and knowledge upto date.

Many women only get breast feeding support from midwives, antenatal and postnatal who don't have additional training.

All midwives are qualified to provide good breastfeeding support

But additional training helps immensely

On-line I think the role is more a signposting one usually.

Not unless there is significant changes in research.

Because we're used to doing it over the phone already.

Breast feeding support is no longer a key part of midwifery it’s now a luxury and often the roll of health care assistants and peer supporters

As long as you are able to explain and demonstrate confidently then I dont feel any extra training is needed for everyday issues. However I think this is needed in order to support more complex issues

There are so many resources for midwives and women. I don’t think it’s needed

I think when midwives qualify they should have the appropriate knowledge to be able to provide support online and if they don’t feel they do they should either gain extra training/experience or refrain from providing support online.

No. Please explain why not:

Core midwifery skill

I think midwifery covers most areas and I think most midwives are confident in providing all areas of breastfeeding support

I feel my university covers breastfeeding well, & I feel confident in my knowledge without having to take extra training.

we receive updates as part of our CPD

All midwives should be / become experts in giving breastfeeding advice

Would depend on individual but one would expect all midwives to be able to provide breastfeeding support whether on or off line.

Our training and Continued training means we are always upto date with the most recent research

It is part of our daily job

Basic support through the early days of breastfeeding and later can often be effectively provided by peer support mothers. However, more complex or unresolved issues need more training / experience in the field.

I'm an experienced midwife and can give breastfeeding advice online as required

My midwifery degree has been geared towards achieving the university bbf accreditation in our final year. I actually think it's been a distraction from other important issues such as pelvic floor and sexual health. That said, we have a lot of breastfeeding knowledge and skills. I would actually rather see less focus on breastfeeding as an undergraduate and more focus on mental health, sexual health, perineal knowledge and for higher level breastfeeding knowledge to be a postgrad course like NIPE.

This should not be required if students have had adequate exposure and thorough assessment, and midwives keep them self up to date with training and experience.

I do not think women benefit from non personal contact

My trust is bfi and we have all had additional training for this. Our role is to provide support for breastfeeding as midwives and if we are adhering to bfi then we shouldn't need extra training.

I dont think it is needed but would be desirable

All midwives support breastfeeding as part of their role and some from personal experience which can help some mothers

I have lots of knowledge from my own experiences, research and time at bf groups and following social media bf groups but no official qualifications

Breastfeeding is covered annually in mandatory training in my trust. I think training would be needed on how to provide support effectively online but not for the actual breastfeeding

Feel confident I could do it but would also welcome further training if available

Part if a Midwife's role

It is integral to midwifery role.

I think it's a skill that would come with experience of trouble shooting breastfeeding issues, something training cannot suppoy

No. Please explain why not:

Training doesn’t always make you an appropriate person. There are some great members of staff with no training, and some awful staff with loads of training.

Intrinsic trouble of the Midwife

Because the principles are the same however the information is provided

I feel as a first year student midwife breast feeding support for most women is very easy to give and as long as a midwife had patience and kindness (as she should) it should be successful.

As we a taught in university at the BFI standard and also have OSCE examinations to demonstrate competence in the skill.

All midwives should be proficient in providing BF support!

Personally during my training in a BFI accredited trust, I have had loads of breastfeeding support experience. I can't see how doing that via social media should be any different. Video chat could be used for physical demonstration or pictures could be used if video is unavailable.

We should have the knowledge and experience to answer questions in any format

The theory of breast feeding is the same

All midwives are trained to provide breastfeeding info, and all hospitals I know of require regular updates for all midwives. I think additional training is most useful for lactation consultants etc who would provide very in-depth support in difficult situations. Otherwise general knowledge gathered during training and updates should be good enough to provide support to the majority of women.

The advice will be the same whether over the phone or online

I have gained a wide range of breastfeeding support advice from working with women and babies in hospital. Also speaking to breastfeeding specialists.

The midwifery training in itself is enough training to provide support to women

Online doesn’t change the guidance we provide. It’s just provided in a different way, the information isn’t different

It should be done in person for better advice

If have learned adequate hands off teaching and troubleshooting during training, should be sufficient

It is included in a midwives role. If the midwife wishes to provide support but feels that they need additional training then it is up to them to seek it on an individual basis.

Breastfeeding training given as a student and a qualified midwife

Midwives are trained in providing the support, so it would be okay online too, it doesnt make the advice any different

as long as you are UNiCEF trained, you should be able to give advice online

So much rubbish is shared by those with no qualifications at all that, although additional training might be beneficial, even the most basic of midwifery knowledge is better than what a lot of women are getting

No. Please explain why not:

We should all always being giving the same advice to women

All midwives are trained in breastfeeding support as part of their training. All midwives should be able to support a woman to breastfeed. It should not be a specialsit skill. If midwives need more training, then this signifies a problem with their undergraduate training.

Should be provided at trust level

Personal experience

Providing skills are maintained via continuously professional development, I do not see why a Midwife should need additional BF skills to support women via social media

It should be included as part of RM training- mine was. Albeit not via SM

Have regular in-house updates

Lots of BF content in university modules and in practice too

Should be include in midwifery training. Better to visit women face to face to offer this support

With the all the training midwives do to do their day to day why is online any different

We are midwives and with that we are taught how to deal with breastfeeding issues

Experienced midwives who have sound knowledge can give great advice. However those with extra training have more resources to provide clearer help

I think videos and diagrams etc are really useful online resources and wouldn't require additional training

I think the BFI training is enough

You are able to video chat with the mother to enable yourself to see how breastfeeding is going

Midwives do not need extra training to give advice oine or off it

i dont think so .. but i think update training should be provided

It is a mandatory requirement of our training and personal development throughout the year

We are fully qualified in BF support at the point of qualifcation and know how to access up-to-date guidance

I believe the Mandatory training has been enough since qualifying

There is a strong focus on breastfeeding in midwifery training so I think there wouldn’t be a need for additional training, maybe refresher training available if midwives feel they need it

Skills should be engrained through standard training

You get a lot of breastfeeding experience as a student midwife

No. Please explain why not:

It would be beneficial however not completely necessary as during the midwifery university course we have in-depth knowledge surrounding overcoming problems

I have had excellent breastfeeding training at my Trust, which could be applied to online support

Not needed, but would be beneficial

I feel that you get good training over 3 years plus my trust do a yearly mandatory update for breastfeeding

Because I tend to signpost rather than give specific advice

I think it is important to be able to articulate the information we learn throughout our training and in yearly trust updates. We can then sign post to specialists if necessary.

I think the training we receive in hospital is adequate

I feel that a great deal of our degree is highly informative on breastfeeding. We have exams and Essays on breast feeding and have amazing lectures too. I feel more than confident helping and supporting women to feed

Pre registration is appropriate enough to offer advive + support

We have this included in the degree

The midwifery degree teaches substantial amounts on breastfeeding (spoken from personal experience from my own university)

I have been trained using unicef BFI and if this is not considered effective enough to support new mothers midwives should be retrained to do so. I feel often breastfeeding specialist cash in on new mothers at a vulnerable moment in their lives. I do wish midwives were able to spend more time with women to help but unfortunately the time restraints make it impossible sometimes

I don't see the difference is what training there is in relation to giving breastfeeding on the phone in difference to online, the advice is the same the channels of communication is the only difference

In university we covered a lot of breastfeeding and supporting women,. We learn from them as they do us as well as evolving research. I don't think there is a need to have extra breastfeeding qualifications

Surely it is part of the role of a midwife, that support should be no different than face to face interaction.

I believe it is something you learn through practice.

I do not feel additional training is needed however feel midwives should be updated regularly to ensure they are giving out up to date evidence based information.

Many of the principles of breastfeeding can be communicated in person or onlibe

Breastfeeding is well taught at universities that are BFI accredited and with experience in midwifery it is easy to gain lots of experience in breastfeeding support.

If it is someone's passion they will be good at this.

No. Please explain why not:

It is a fundamental skill of a midwife . The culture surrounding its importance needs to be addressed in training by universities and hospitals that take student midwifery.
Covered in training
enough experience and updating regularly as self regulation
I think the BFPS training is adequate for what we require. But the understanding of when to refer and escalate is crucial.
All midwives should be competent at providing simple BF support
It’s not that difficult
Dependent on training whilst a student
The degree & work experience is sufficient.
I would how that they would feel comfortable to be able to provide women with breastfeeding support and learn as they go.
I am a member of various fb groups for personal use like LLL and extended breastfeeding support so kinda know how things work
Lots of support is within the scope of midwifery practice. Signposting is important when beyond that scope.
You do it in practice constantly so there isn’t much new info to leern

Q17_2_TEXT - Yes. Please explain why:

Yes. Please explain why:
For practical teaching
Yes absolutely, to ABM counsellor standard as a minimum
Midwifery breastfeeding training can be quite basic and annual updates vary.
It's probably more important to have this training as it's harder to communicate online
I think more breastfeeding training is needed in general but with online support in professional boundaries will be needed also.
It is different supporting someone online to in person.
any support online should need further training, due to losing the face-face interaction
Advice and guidance changes, good to keep up to date, refresh use of correct and up to date online tools
It is important to be factual and sensitive when providing online support

Yes. Please explain why:

Different set of language skills required as cannot demonstrate as simply

We have regular breastfeeding training within trust mandatory days, as with all midwifery skills it is important to keep these updated

Bf has been taught in 1st year with the view of an OSCE although this has been cancelled due to COVID. I know bf is taught and support practiced in role plays but there are no more PN specific placements for the degree duration

To ensure that advice is up to date and BFI appropriate

Keeping up to date with Public Health recommendations is key.

Will need to be able to go into great depths to explain how to overcome obstacles

I think there is a lack of honesty of just what it is like to breastfeed and how some things midwives say are not normal are actually normal such as sore nipples in the early days

It’s easy to give opinions rather than facts. Many women say everyone tells them a different way to do things and this confuses them

Breastfeeding is a complex topic and whilst basic support can be offered I feel it would be beneficial to have further training to help with some of the complex issues mum's face

Midwifery education in relation to breastfeeding is very limited and when women seeking support are asking complex questions about older babies I am not confident that all Midwives (myself included) would be able to support them appropriately. For the more basic first days/weeks queries I believe any midwife could confidently support women online.

Additional training would be up to date and the information would be more accurate

Only in terms of tips or advice in order to deliver this support online

The information needs to be evidence based, upto date, in line with your Trust and everyone needs to be providing the same information for women to prevent confusion or mixed messages.

Not enough breastfeeding education and practical experience is mandatory in midwifery training.

More support is needed to new mums, breast feeding support is often left to the MCA but if they aren't on shift mum is set up for failure before she even tries and some of the midwives lack experience and time

Midwifery training does not include enough information on problem solving of Lactation issues - it’s very basic & you need far more than this to problem solve on line

provide resources

Can be really difficult to assess bf online

to keep upto date wkth current guidance and advice

Training yes as it means the information being provided to women is evidence based and correct and not impacting on their breastfeeding journey but as for experience no. I’m a student who does not have a child and has not breastfed yet am able to provide breastfeeding support without experience.

Yes. Please explain why:

Because many mothers are recieving poor unsafe and incorrect information.

Trained professionals who are constantly maintaining their qualification have the newest breastfeeding evidence and support and are un a better place to offer women support

It requires a certain skill set and knowledge particularly about safety and professionalism online

It is difficult to provide bf advice when not face to face. And any format that is not our usual should be considered for additional training.

We already promote this and have attend anywhere breastfeeding virtual support that runs 3 times a week, we also have a specialist breastfeeding team that support challenging cases only in the community

Regular updates

Online support differs from face-to-face interactions

Not in all cases but can be helpful

Recognise further problems

The training I got as a student and newly qualified nhs midwife was so basic it was almost useless.

In some situations yes absolutely. I don't think anecdotal evidence is always beneficial and I think midwifery input is required, whether that be training peers or providing care directly. Infant feeding is a midwifery speciality, it's complex and can result in neonatal health issues of advice isn't given correctly or issues not picked up on

And face to face

If its via video it would be OK but I think virtual support with only text could be difficult

Providing virtual support is different to face to face so different skills need to be learnt through training

All midwives who provide such support should be BFI trained or a breastfeeding consultant

I believe breastfeeding support is one of the areas in which Midwifery care in the UK lacks greatly. Improving midwives knowledge of breastfeeding and how to support women would not only improve women's experience of breastfeeding within maternity care but also attempt to change the essence of conversation regarding breastfeeding amongst midwives. In turn perhaps increasing the breastfeeding rate.

I think the updates that we receive from the TRUST are enought to support most women, but it is necessary to have at least someone especialized in that field for the most complex cases.

Most midwives have basic training so advance to training would benefit online support.

To enhance the support that can be offered

All midwives should have further training in feeding to support women

Training and relevant information is essential. I have learnt so much in the last 10trs More than I knew when I fed my own babies

Addition training is always beneficial

Yes. Please explain why:

Explain things better with current reseach

I think it is important for all midwives and hcsw

Lactation Consultant as a minimum. I have seen too much 'advice' from poorly trained members of the public

It can be more difficult to assess breastfeeding via the internet as opposed to face to face- we may need extra skills to be able to help women.

Breastfeeding is complex and women need Face to face support as well as online/telephone support many women during Covid 19 feel isolated and unsupported so contact with professionals needs to be timely and where the woman’s needs can be met and she can be sign posted to relevant care.

Local tongue tie professionals. Connections with Health visitor etc

I believe you need to be able to ensure full in depth knowledge is provided to women

Access to a range of external support groups, understanding of different cultural/psychosocial barriers etc

To be able to provide the most up to date information

BF support, in my experience, is quite poor from midwives, particularly older midwives. Keeping up to date training for any purpose is beneficial and the more reasons to do so are advantageous to women and babies.

To provide the best care, more training needs to be taken

For best practice and up to date in statistics,

We have a family and babies support group

It can be difficult without being face to face

because it is easily accessible for most women

It is vital to be able to communicate this effectively and it's even more difficult online. The topic is emotive and it is crucial not to get involved with using terms such as Fed Is Best.

Because you have a better understanding

BF training during the undergraduate degree is rudimentary

Providing g specialist expertise and covered by indemnity insurance

For additional resources

It is important to be sharing consistent information but also being able to respond appropriately to specific issues women may be facing. Having access to resources that are in line with hospital policies that are also professional and informative.

It is hard to provide info on line, without facial cues and seeing cues from baby. It would be beneficial to have a short training session on how to support from afar!

Yes. Please explain why:

To ensure upto date and user friendly advice is given

Face to face is so much easier.

I imagine it may be difficult to complete assessments and provide recommendations without seeing the mother and baby in person

To ensure it is completed safely and appropriately

i think that midwives need to be confident in providing one to one bf care and if so, should carry on. those that are not confident, could partake in extra bf sessions to provide support

Online support is very different to face to face observation and discussion. Resources to share with women also are needed

In some circumstances yes women may have specific needs such as need for tongue tie assessment which I would not be comfortable undertaking due to lack of experience

It's an important part of our role and we need time and expertise to be able to support women Nd their families with feeding choices and options

to esure women are provided with up to date evidence based information

Could be helpful in situations like covid

Midwifery knowledge about breastfeeding is essentially a basic introduction. Some queries online will be basic but some require a far more advanced knowledge base.

Because it is such a practical subject. Not currently being done very well. I do 1:1 or prior to covid ran workshops

Yes. So important did Midwives to understand and unpack their feelings around infant feeding and breastfeeding in particular. Having up to date safe infuriation is key

It's a very different medium and harder to communicate effectively

Not everyone is confident in supporting breastfeeding or troubleshooting and professionals de-skill

I think support in using social media appropriately, whilst giving advice requires training.

It would depend how much UNICEF training was available through midwifery training, but this is not as in depth as training with other breastfeeding training providers such as LLL, NCT, ABM or BfN

As a student, although we have an abundance of time for providing beautiful breastfeeding support, like with any skill or knowledge, we often acquire this along our lifelong journeys through our careers. And i do feel the more experience, more discussions with other professionals and extra study days will hugely benefit. However, i do feel it will differ between each person and their confidence in giving breastfeeding support over an online forum.

There is no requirement to support x number of BF women (in the same way they have to support 40 births for example) and so I note many student MWs skim over this element and do not reflect on the impact etc. This means a PostReg training programme will help midwives when they finally realise how important the knowledge is.

Additional education would provide midwives with better assessment skills and knowledge of troubleshooting breastfeeding issues. Mismanagement can lead to breastfeeding cessation or issues with faltering weight and psychological repercussions for the mother.

Yes. Please explain why:

I think breastfeeding support in general is woeful and so many issues are missed or belittled.

As this pandemic has shown many families have relied on online support and HCP's should be confident in dealing with common queries and also offering further support (virtual or in person) for complicated issues

keep updated

BECAUSE BREASFEEDING RATES ARE LOW IN MY AREA THEREFORE THE SKILL CAN BE LOST SO BY ADDITIONAL SUPPORT AND TRAINING WE CAN KEEP OUR SKILLS ALIVE

As written support can be harder to interpret than face to face support. I also feel it's less personal and women may feel like just a number rather than an individual. Professional practitioners also need to be certain that they are providing evidence based upto date information.

Different resources required

I believe experience or training would ingrain the knowledge. You can then approach situations in a more abstract way and know how to guide using words rather than relying on actions. A telephone or text conversation is far more challenging than a face to face visit.

For midwives to have the confidence to relay the information online in a clear concise useful way

consideration has to be given to the alternative medium

Probably to ensure that we have picked up the correct advice in our training. Unfortunately in midwifery there is potential to pick up a lot of bad habits!

Updates always important, including local referral pathwats

There are nuances in face to face meetings that are missed in written communication. This risk could be mitigated with more training

Training in confidentiallity and online safety may be appropriate

To help mothers

Often BF support requires 24/7 support so social media use can help facilitate that. Some women may have to wait for Appointments / support from health professionals so Facebook could provide a quicker response.

It's easy to support feeding face to face using aids, but difficult to explain over a phone

It has worked well during Covid.

You need ecperience and confidence to be able to deliver education/ support when you cannot physically see whats happening

It can be different explaining in person rather than through text or online

i don't think the training provided as a midwife is sufficient

our training involves a lot of gesturing with dolls and knitted boobs. extra training to make sure the replacement communication is clear

To give more in depth information with women who may have complex questions

Yes. Please explain why:

Perhaps some advice on how to use the media via it support or how to advise parents to hands free bf advice

Learning new way to communicate effectively to show and explain breastfeeding over the internet would be a skill that I think I would need training in

Gives further credence, info is often mixed and with additional training/qualifications you are more likely to keep up to date

To give women same info consistent etc

i mean experience is needed to provide breastfeeding such as midwife

Information changes continuously, and your knowledge is only as good as your initial teacher/self guided study. I did a 2 day study day which was compulsory when I started at my trust and I felt very well informed for a year or two. But due to high activity of my unit, I have not been able to dedicate much time to breastfeeding whilst in practice as there's almost always overriding clinical need. As such I don't feel as well informed and often now defer to others as I don't feel I am knowledgeable or skilled in providing support

breastfeeding training as a student is inadequate

Little hints and tips not always taught....you learn through your experience, and it helps,with supporting women

New research is always emerging so it's important to keep up to date with skills and knowledge

The more education the better the service!

It given you a better understanding of the processed

Annual updates

As students we are taught breastfeeding support and have experience of it so that by the time we qualify we are able to give BF support and rectify many of the problems that women experience with BFing in the early days.

To ensure correct language is used and advice being given is the same by all trusts to avoid confusion

Not everyone has same experience in training and may need more training to do this effectively. The knowledge and experience of our midwives are as varied as the people we support!

To ensure women get the same advice eg following BFI Standards

There is a frightening amount of misinformation coming from midwives online

It may be different than supporting someone in person

Specialist subject

To provide correct and up to date information

we have an update yearly consisting of 2 days. It is important that all health professionals offer consistent advice and support.

Yes. Please explain why:

I believe having some practical experience if supporting women to bf is essential. Additional qualifications aren't always easy to access owing to cost/timing. I believe training in using the online platform would be essential including having algorithms to support the professionals if a problem was identified to ensure consistent evidence-based advice is given.

Any midwife practicing on social media requires social media training and a professional profile

Social media is constantly evolving so the practitioner need to be able to be confident in things such as sharing and privacy in order to safeguard women's information and dignity in a way that you would not have to consider if this was being done face to face

Msc Midwifery in teaching

Some people are very poor at communicating over typed messages. Offence is easily taken, and midwives also need to ensure they are not working overtime on women who request long and frequent support sessions

Initial newborn is covered by midwifery training however infant feeding beyond the first few weeks is not. The older baby and child have different feeding requirements and concerns by parents

In order to ask the correct questions online to mothers to build a holistic clinical picture.

It is very difficult to explain breastfeeding voa text. There would need to be some standardised advise as well as the ability to change it

We are trained in face to face support so I think it would be beneficial to having training in how to do so without being present with the woman and baby.

So we all give the same advise to current standards

Where I trained we had this and it goes hand in had with BFI, I understand lots of universities do not offer this and it's important to be aware of, although all the intervention in hospital with a large majority of births makes it near impossible to succeed or get off to a good start

Depends on the age of the child. I'd be confident to support newborns but if there are continued feeding problems past two weeks of age I'd struggle

Mandatory training annually is very important

The midwifery degree does not equip you for the nature of difficulties women face with breastfeeding. Own research and /or additional study is needed.

I have heard of very recent incident when my friend gave birth in hospital and another mother was asking for help with breastfeeding and a midwife told her to just give fornula instead of helping her find a solution. And this is a very common thing just after giving birth. The hospital has BF accreditation which is even more frustrating. which

Would be idea to provide and promote breastfeeding support online, especially as sometimes women just have quick questions and rather than awaiting the next visit from the midwife or health visitor they could quickly just post their question

I feel there is still alot of mixed messages about breastfeeding and out of date beliefs and information amongst midwives.

Because there is a skill to bring able to give information remotely

To give the most up to date advice rather than hearsay advice based on just experience

Providing virtual support is different to face to face and so training in how to overcome barriers in virtual care delivery should be included

Yes. Please explain why:

i think it will help to have specialist training with how to advise remotely.

Things change, I haven't had any bf training since qualifying and worry I may not give current advice

It is someimes difficult to support over the phone particularly if a woman's first language isn't English or if she has a leaning difficulty or disability.

May need help phrasing things in an accessible way to ensure women understand

Providing support online is very different than in person and therefore, more training would be required for this to be done effectively

Doesn’t matter if online or in person correct information is needed to be given

All breastfeeding training should be continually updated

definitely, so much conflicting information is being given, confuses mothers, who often turn to bottle

Updates are needed to prevent desensitisation and just 'putting the baby on' because of time restraints to get the woman off the ward

Sometimes it can be forgotten

I think it would be valuable if new guidance comes into place and to refresh what is already known to insure the best support is given. It may be harder to give support online so we may need to adapt to that as I feel it would be easier to given guidance and support in person

It is important to be providing woman with the correct information

Ways to explain in online rather than in person, might be easier making videos

To cement knowledge and to spend more time learning about communities, problems and what women really want and need from us

It is hard to give correct advice when you can not see what’s going on! It would be naive to assume all people using Facebook for your services understand technology so many struggle to interpret your advice.

to keep upto date with current guidelines and advice.

To give information of high quality

Not all individuals are competent using technology and it can be more challenging to provide support remotely so reliving information in this way could be developed to deliver it efficiently and clearly in this manner.

To optimise support given online as it is different than being face toface

There is just as much misinformation as information on facebook, women need to be signposted to official verified groups

Its harder to provide instruction/information online as it could be interpreted the wrong way so people need to be taught how to express themselves online appropriately

Text books only explain some things, experience teaches you deeper.

To keep up to date with modern methods

Yes. Please explain why:

To avoid giving poor/ unhelpful advice

Our trust is BFI accredited and students don’t cover enough of this in their training

I think that all support online which can be seen by many women should be BFI accredited where some trusts are not.

It's different to helping in person, may need to use different techniques to support women.

Experience working alongside mothers wishing to bf after qualifying gives you more tools to provide care.

To understand how to problem solve and know previous problems

skills are important to understand what the issues are and IBCLC give you expertise knowledge

Many universities do not adequately prepare students. Completion of preceptorship and local training (as a minimum) should be essential.

Sometimes it can be more difficult to communicate online than face to face communication

There is inadequate bf training as part of degree training imo.

Most breastfeeding support provided online is relatively simple, positioning & attachment normal newborn/infant behaviour. But it needs to be backed up by access to knowledgeable experienced one to one support

To be able to provide detailed information that allows women to make informed choices, the more knowledge the better.

Training does not give you the knowledge to provide a higher level of support necessary for some breastfeeding mums

For further knowledge when women are breastfeeding a baby who is over one month old

Bf knowledge as a midwife is limited. But especially limited beyond the initial 10 days. Very limited knowledge on supporting TT and other hurdles that come up beyond the immediate PN period

Teach women how to spot information that perhaps isn’t evidence based

To be able adapt knowledge or training so it can be delivered online.

As midwives we have minimal training in supporting women in person, we would need additional support to do this online

It's different from seeing someone breast feed in person. You need to have training and skills to be able to provide information that makes sense over video or a post and understand how to give support when you don't see that person or how that baby is feeding. You can't see the sucks and swallows so you need to be clear how the women will identify that without you being able to show her in person

Only on the basis of learning technology and how to provide the best support on different platforms. Not necessarily extra breastfeeding training

Confidence building with using online forums would benefit me. I have worked in bfi accredited units and had good training/updates yearly so felt confident with bf and giving good advice, however I now work in a unit that isn't bfi, doesn't have an infant feeding lead and has not provided infant feeding training. The difference in the quality of advice the women receive is stark.

No one size fits all for breastfeeding, it’s often experience of lots of options which assists women.

Yes. Please explain why:

Trust Specific Traning

Explaining something face to face I think is easier as you can guage from reactions whether somebody has understood what you are saying. Additionally extra training may help to improve the wording used to ensure clear advice is given.

I gained a lot from the training and it gave me confidence in BF support

How to convey the information in a way which will be correctly I'm understood by women via social media

Everyone should receive the same training so same levels of care can be provided.

To broaden the depth of breastfeeding knowledge

because the more knowledge about BF the better we can support women

I think it should be a specialist role so advice is given by a small amount of people to avoid conflicting information.

It is not required, but helps.....I have a long specific interest in BF support.

to improve the experience and duration of breastfeeding

Giving advice online requires more skill than can be gained as part of the pre-reg qualification.

To ensure that the way the information is being displayed is appropriate and is relayed correctly in an online format

But not outside of local trust training, although some additional training on breast feeding problems would be beneficial

Additional training is definitely needed to support online

Our BFI training helps inform us of the physiology and being taught by LLBC midwives gives us lots of tips and tricks

You don't get all the necessary training during undergraduate degree.

Although breastfeeding is covered in university, having more training on providing online support would certainly be beneficial to ensure it is being conducted effectively.

To be completely up to date

So that the advice given is current and evidence based

I think there should be regular updates as new research comes about and keeping midwives up to date enables the best support to be provided in the best manner.

To avoid confusion or mixed messages, varying degrees of experience and knowledge, anecdotal evidence etc

I think it's much easier to describe something and support a woman when you are with her and can move your body/use gestures to help her. Doing things online can be alot harder

Yes. Please explain why:

Maybe minor training as providing support online is completely different to providing support in person so experience may be needed for midwives to adjust to this.

Need to be up to date

In general I have found that midwives despite supposed to be able to support women to breastfeed, don't always have up to date information, inconsistent advice and don't have the time to sit and help a struggling mother.

Evidence based practice

To learn different techniques in how to observe feeds, demonstrate etc.

There is so little taught on how to spend time offering Mother's one to one time on breastfeeding. Latching, positions, normal newborn feeding behaviour (cluster feeding) could all be explored further for midwives to relate to mothers.

Communicating information online is very different to in person and I think techniques should be talk to make sure the correct information is given online

May help to consider how to take a history, triage and give advice in written format.

Providing online support is not taught at uni

It would be good to have breastfeeding videos/classes/support online, but there is always the option of getting support from local midwives/birth centres/ postnatal wards to ring or go to at any time (not currently with vivid)

Extra support needed online in the PN period

Keeping up to date with training is important, things change so often. And you would need to learn how to come across in a professional manner.

I feel breastfeeding support isn't offered as much as it should be

To give us confidence to ensure we are giving correct advice.

We are not taught to give information over the internet. I would want to be confident in how the information should be shared, I would also be worried about not been able to give tailored advice about technique etc

my training hospital was not BFI accredited and I feel there is a lot more that I still have to learn about BF support

Having breast fed my own baby for 15 months, I believe a lot of the information I provided to women wanting to breast feed before I had my own baby was very misleading, naive and unrealistic. This information was always research based and what I had been taught in university and on training days as a qualified midwife but was never of much use to me when breast feeding myself. Support needs to be realistic and beneficial to mothers

As a midwife I am very co code to in providing breastfeeding support for mothers. I can recognise why some mothers have difficulties breastfeeding and can help rectify this, however I do not have the knowledge of the specialist breastfeeding midwives regarding lactation etc

I think on a personal level, being able to decipher from words what help someone really requires is a talent and would require additional training. Some may present a problem in one way but when looking further into it, other issues may be discovered. I also believe it is important to be able to provide correct information in a way that is tailored and individualised

Yes. Please explain why:

Although this is a tricky one, I think that if you understand how to use online platforms and are up to date with support knowledge then possibly not but feel that it would benefit those who are less confident online or with their knowledge.

Experience is needed to offer useful advice

Because you need to be able to be flexible and succinct on a Facebookplatform, so perhaps training in the actual “teaching” or presentation of breastfeeding

I think to be able to explain breastfeeding online the person needs to be an expert in the topic. This would enable support in all areas & questions posed.

To provide support online i feel that professionals should undergo training on esafety, the risks of social media and how to recognise issues of concern regarding the woman’s wellbeing

Able to identify and support more complex issues. Be able to justify decisions and referrals with robust criteria. Able to support mothers to not assume there is always a TT issue

For consistent evidence based information

Women seek further information than what is provided by routine midwife care, therefore specialist knowledge/ experience is an advantage

Additional training would benefit midwives in providing online support for women as previously support has usually been within womens’ homes or in a community setting face to face. The same information would obviously be given but training on a way to give that information via online networks would benefit ways in which to demonstrate information in a clear way in which all women can understand

Breastfeeding mothers are somewhat supported in a hospital setting and for some time at home, but not nearly sufficiently enough

I think it helps to have deeper knowledge to get to the issue more quickly

I’m compliance with UNICEF BFI and the WHO Code of marketing

Evidence changes.

y

Q24 - Please describe any additional training or support you feel would benefit you and/or other midwives in using Facebook to support mothers:

Please describe any additional training or support you feel would benefit y...
Sessions on the above to make midwives aware of the pros & cons
Clear guidance from NMC and trusts. Clear management plan of any adverse comments from women. A few women on the local Maternity Voices Partnership have made negative comments about individual midwives and units and as professionals we don't seem to have any right to reply or defend ourselves, our organisation or profession. Whilst I uphold to right if women to question their care and seek a response doing this through social media is not only inappropriate it may frighten other women and prevent them seeking professional support or opinion. Maternity Voices Partnership needs monitoring and the users reminded it is a forum to share ideas, promote maternity services and inform not name and shame midwives and organisations .
I wouldn't want any training in this matter
Some training on use of social media in a professional capacity. Policies tend to err on the side of caution and just focus on not using it.
Guidelines in terms of creating and maintaining boundaries so that women understand what to expect from this service and to maintain professionalism but also so that it does not become overwhelming for midwives (so they don't feel they have to be active on the group all the time) Confidentiality
I would suggest that any midwife wishing to provide online Lactation / breastfeeding support should have an additional qualification equivalent to BF counsellor status Midwifery training simply does not cover Lactation issues adequately for midwives to provide such an on line service safely
I wouldn't want to do this at all
N/A
Not sure
Any of the above
Definitely additional social media and digital skills training should you wish to take part in monitoring or being a moderator of the social media groups.
I was investigated for raising a public concern online. The public concern is still damaging babies. I have used social media to educate + empower women since 2012. I started a page when I was to told be quiet about my chosen subject by my employees. My social media concern ruined my career as a midwife after 30 years. I didn't do anything wrong but was told I had breached guidelines. I hadn't at all. It was a case of bullying by a trust which had bullied me for years, they used this opportunity to further intimidate me, the RCM supported them rather than the concern + joined in the bullying. The page and the education still exist. I'm a nurse advisor now. The guidelines didn't help me at all. I didn't breach them but I was slaughtered. Midwives should be made aware this can happen.
Uni advice was do not use social media. They had no idea of its potential usefulness. Most trust-based maternity groups are awful, just happy clappy PR.
I don't think we should move to an online presence. The population spend far too much time online and looking at screens. This would only increase that and increase the need to instant gratification. The expectations would become too high, placing yet more stress on midwives
Clarification on situations which can be supported online and which require assessment in person. Understanding where this fits into the already hectic life of the midwife - who would fund it, what would the hours be? Will midwives be paid for their time?

Please describe any additional training or support you feel would benefit y...

I think we need to move away from the assumption social media=\=unprofessional, and all the training I've had or discussed with others have been about protecting yourself and defensiveness, not about safe usage or recommendations

Thinking about my specialist area; perinatal mental health; I'd like support and guidance on how to safely offer Facebook support to Mum's given the risks this client group can sometimes present with.

I would not mind participating as a midwife in a group that represents the trust or my unit, but I would never feel comfortable using my personal account, with my personal details on it to give advice on the social media, for several reasons, but mainly for privacy.

Dedicated e midwives

De-escalating tricky conversation

I follow several support groups. I haven't commented because some participants are aggressive. With no moderator, it's difficult.

A SOP was developed and plan for posts in conjunction with the Trust communications team.

I do not plan to do this and will fight against it all the way

.

None - but Facebook itself is not a universally moral or honest platform provider - this influences the debate

Direct contact with allocated midwife to avoid confusion with conflicting advice.

I feel there should be incredibly strict monitoring of this and it should never be from personal accounts. Any advice given by midwives can and will be screenshotted, edited, shared on women's personal accounts, and has huge potential personal risk to midwives. I have seen many midwives berated and destroyed on social media, comments taken out of context, interactions I have witnessed and been present as the midwife posted, knowing background from their in person consultations and the woman's medical notes - and yet the woman has publicly posted very different information. I don't think any interactions should be public.

Knowing what is appropriate to share and what is not. Having dedicated time to spend to creating posts and posting them. Having clear channels of communication with specialist midwives to share useful information for women. Amy of the training listed above would be useful

Clearer support from NMC

local support and guidelines.

women have felt a little put out during this pandemic and I have noticed a lot of activity on a local hb website for feeding. it has been great to watch others peer support each other and midwives to continue with continuity that otherwise could have been lost

Confidentiality and guidelines of what can be discussed on a social media forum.

Trusts should offer an online professionalism training and guidance

Interactive training with clear guidance and framework for use

Clear information on what is you should/shouldn't do when supporting women by using Facebook

Please describe any additional training or support you feel would benefit y...

I took early retirement from my band 7 role as all I felt I was punching child potection info into the computer with very poor training. The role I loved was rapidly being eroded and taken up with support workers. I decided to take control and have a lovely wee clinic. I uses facebook to book, sometimes post educational messages.

Regular bf training and supervision, clear guidelines and boundaries plus allocated work hours. It training. Potential for virtual as well as written support

I have knowledge from a previous career, so am aware of some of the behind the scenes elements of social media. I feel like with the development of any new technology midwives should be aware of what is available and how best to use it.

Clear guidance on boundaries. Continued development on interpreting the evidence.

I think there is a risk that midwives will let work bleed into their personal time and so there should be strict guidance about midwives switching off from "professional" FB when they are not working.

Local guidance and training from trust is imperative to support confidential, management of boundaries, regulate information provided and monitor safe practice. I think that the use of Facebook should be under the auspices of the employer to support the midwife to help benefit women and their families. Is there any guidance on complaint and liability and is the midwife covered by the employers indemnity insurance. I have worked as a midwifery expert in medico legal clinical negligence cases so have extensive experience in this field

Obtaining evidence based information and evaluation of the findings.

Online awareness training

Digital media training would be helpful

This should be built in to midwifery degrees! Our entire lives are online for heavens sake! Midwifery programmes have themselves gone online during lockdown and they've no excuse for not providing thorough and up to date digital training. All NQM should leave university feeling competent with engaging professionally online in any capacity, be in professionally on LinkedIn, setting up influencer accounts on twitter (Milli Hill, Rachel Reed, Sara Wickham) or supporting women as part of our midwifery role.

Training managers/senior midwives to increase awareness of the use of social media to support mothers would be beneficial, as this would likely lead to wider services being offered to maternity service users. Our midwives who provide digital support services collectively have a separate support group run by myself and another mentor so additional guidance and support can be offered to each other,

Any training would be welcomed. It is a world that we are just expected to know about, how we communicate on line and in person are two very different scenarios.

I think a code of conduct should be applied to any social media groups and everyone should abide by these.

The only training I've received was basically - beware social media! Yet it is an essential part of my pob in terms of keeping up with research (Twitter), communicating with colleagues on service improvement/policy (Facebook) and communicating with women (Facebook, WhatsApp, Zoom). By the way, the Spanish group I support as a midwife is a WhatsApp group, not Facebook - I feel I would need more tech training to set up a Facebook group. I received (independently) some great zoom training, focusing on using the interactive tools and building group cohesion on line - essential if we are not going to just use these tools didactically.

Trust guidelines and guidance on how to manage supporting mother's and staying within professional boundaries

Knowing about training courses on certain topics eg still births

Facewives training

Extensive training to ensure the safety and protection of both the women and the midwives.

Please describe any additional training or support you feel would benefit y...

I'm older so FB does not come naturally. Basic skills would have been useful, ihave had to teach myself Set of guidelines should have been useful. A dummy FB page would have been good to practice on

I think social media is a great platform when used well, as of yet I'm not aware of any support groups which are run on social media in my Trust.

How to protect your own privacy

It would be nice to see some guidance about what I am and am not allowed to do online in terms of support.

As a midwife, a midwife should perfect himself so not only facebook, every place to support every mother

Ensuring that there was a set time to take a break

Unsure if I feel it is strictly necessary; I feel it's a very complex subject and most of those complexities aren't due to lack of training or need for training but rather regional/trust based guidelines/difference in services provided/capacity for certain services/wealth/experience of practitioners within trust (I.e. supporting breech, VBAC, etc), and personal opinion of differing midwives.

Clear. Arizona's and trust guidance would be helpful

The above as haven't received any

Support from trust I work in to set this up and understand its place would be the best place to start for me.

It would have to be a generic health board account.

Basic IT skills, conflict resolution, understanding of online etiquette and trends (e.g. spotting/ deali ng with trolls), how to combat misinformation through facts and deescalation.

It'd be interesting to undertake but I'm unsure how comfortable I would be recommending Facebook support groups

Unsure

Part of my role is creating professional training for midwives using social media which I have undertaken at the 12 NHS Trusts Facemums has provided care in. This level of training is essential to ensure midwives have the evidence base from their Trust and professional body to ensure safe care and professional practice. Additionally all midwives I have trained are placed within Facebook groups to enable them to discuss ideas with midwife colleagues and then they can have one to one support with me or my colleague to enable professional confidence and skill for all midwives participating in Facemums

Training in dealing with the nastiness of doulas, women and the pro-trans lobby, who attack us for using words like "women". I use facebook both as an IM and an employed midwife and the nastiness I see on the Trust website from local doulas is shocking. None of the training helps us deal with this, or the nastiness from the woke trans people.

Peer support - shadowing midiwves who reguarly support women on Facebook

Basics how to use FB , some people still don't have an account

digital conflict resolution training.

Looking at the Facebook groups available including mvp it is very easy to get negative comments on social media. This is then always present and linked to the hospital

Please describe any additional training or support you feel would benefit y...

Providing social media training is offered and is to include conflict resolution on social media. Social media can be very vicious and toxic, it can harbour bullying and unpleasantness.

Ceasing of management threatening disciplinary action!

N. A

Conflict management on social media

I feel Facebook as a platform for support and feedback for families is very important our trust is years behind and we have a group for colleagues but not a public forum, I regularly signpost women to local Facebook support groups I feel sharing and hearing experiences is so important to care. I'm thinking about setting up my own zoom classes and o called the RCM for advice and got - zero!!

Maybe a confidential online chat, so women cannot personally go and look that midwife up in their personal social media, they would only know their first name

Midwives should stay off the Internet it is a face to face profession even with the all these groups women are confused and want to see someone

.

Anything that would take fear away from social media use.

A change in guidelines is required for the trust/LMS groups as only allow certain midwives to comment. Heavy restrictions are in place. It puts anyone else off commenting due to previous sanctions of other colleagues

Particularly in areas such as mental health, training to recognise where women would benefit from specialist support in mental health. When working virtually' this is easy to miss.

As qualified midwives we already have to try to complete an avalanche of Health Board e-learning alongside regular supervision for PNMH, SG, midwifery and other professional aspects to maintain compliance for our various roles/skills within midwifery. We barely have time to practice as actual midwives without adding in yet more additional ongoing training. I find I shut off from the RCM offerings of digital training as to be honest I do not either have the time or the inclination currently.

N/A

training and guidance on boundaries. What can/can not be shared etc

Strong and clear health board guidelines on Facebook Groups

Regular training and updated specifically for social media should be focused on as it's only breezed over

?

I think its important to understand the context in which a message is written but I not sure how this could be taught

I would like to understand more about how I can access the benefits of social media without meaning I need to engage in personal use

Knowledge on what to do if someone from overseas contacts you. Where do your professional duties end. I was once contacted by someone from Africa. Less condemnation from employers when responding to women who speak ill of our trust online. I've only ever sought to connect women with services when they cannot reach them, correct misinformation, and reach out when there is clear distress. My employer responded by threatening me with referral to NMC for using Facebook on trust time and misrepresentation of the trust.

Please describe any additional training or support you feel would benefit y...

Training to use social media?- what next?? We live in a snowflake society. If you need training in engaging with people who are looking for information then you are in the wrong profession. Never say anything that you wouldn't say to someone's face. Be kind.

General guidelines on professionalism and self protection

I

I feel that midwives should not be commenting on posts unless they have the correct information and have had some form if training. I dont think midwives should use their own personal profile. There should be a dedicated team who are guided, know how to use social media and can do so by responding as their trust not as themselves.

I think the language we use. How to responsed professiobally when needing to correct misleading information

Training (and access) for midwives to use apps/tools such as Canva & Survey Monkey. Guidance around the importance of correct use of grammar, spelling and punctuation in maintaining public trust and confidence. Teaching of how to use different platforms and how to use the individual features such as Instagram story etc.

Probably some clarity around use of Facebook for support, but we are aware it was implemented in extraordinary circumstances. Our management and communications dept have been very supportive

N/a

Ensuring each midwife involved shares the work our equally and are both fully tech/facebook trained for ease for them and women

Reassurance and clear guidance that if giving evidence-based information this is not inappropriate. Many students/midwives have it drilled in that any talk of "work" online goes against NMC code.

Trust Specific Social Media Training

how to ease conflict online

having clear guidance around boundries and ensuring it is a positive experience for mothers and midwives confidentiality training, clear training on NMC policy

Giving evidence based, individual advice with nuance rather than regurgitating guidelines is something midwifes need to be taught.

it would be useful to have thorough training on the use of facebook to ensure both my own and the women's best interest were being addressed. for management to be on board with using this platform

Plymouth nursing students have a whole module in the first year on online communication and digital skills. They can be found on Twitter under the #PUNC

Inclusive language use Screening links to ensure that they are evidence based

Guidelines for social media use to be properly signposted. Possibly have an alternative professional account to provide support rather than personal account and risk becoming too involved in a persons care and personal life.

The latest evidence based information on breastfeeding and caring for newborns i.e recommendations for dry skin

Basic research skills, list of trusted resources, translation services

Please describe any additional training or support you feel would benefit y...

I think it would need a dedicated team with a senior member to oversee/provide supervision

Introduction to Facebook for any midwives who are unfamiliar

Better social media policy (so vague at present) this needs to be consistent nationally so not misinterpreted.

Training from RCM and a overseas group UKMed. Knowing the boundaries.

Guidelines on the scope of the role, what Advice they are expected to provide and signposting services

none, we are professionals and capable of making wise decisions regarding care via FB.

should midwives use their personal accounts or connect using an alias or admin page. If you use an alias is that against facebook 'bot' guidelines?

.

I think highlighting the importance of moderating information sharing would be helpful, both the appropriateness of what information to respond too and also having the information that is shared being “fact checked” to ensure it is evidence based, correct and appropriate

.

Training in appropriate use of social media. Midwives personal accounts are also available to see once someone “tags” or names them on a post for other individuals in the group to search. Of course most people have privacy settings in place but i feel as though it impedes the work/life balance. A personal facebook account should stay personal, for friends and family only

Any training to insure data protection and a safe environment would be of benefit. Work boundaries or setting up a work account so your not tied to it on down time.

I would like the ability to have a professional/health board profile so that my personal fb would be completely separate. I answer messages on holiday. I'm not sure if any of the above would be useful because it really depends how basic or advanced they would be. I would worry that written guidance would be so tightly drafted as to prevent anything but signposting.

y

Q25 - Midwives and maternity services communicating with mothers and families via social media has become more widespread during the coronavirus pandemic. Please describe any changes you have seen and how you feel about them:

Midwives and maternity services communicating with mothers and families via...
Lots more negatives about what had been communicated
Women are less anxious in terms of knowing what to expect
Sorry only seen negative one. Our local maternity service was one of the first to reintroduce partners at scans and when we announce it the MVP launched into an argument that the Trust communication Dared to use the word patient (Which I agree was inappropriate) . Then the argument grew until it was evident A small number of MVP were even offended by the word “woman” and suggested the trust fund a more “gender Neutral term”.
Popularity has been needed at this time
It has helped giving upto date info re covid an the provision of some care
None
I have seen an increase in people using the facebook page to complain about care. Especially visiting restrictions. They feel it is a 'faceless page'. However we feel we have to repond to all comments which is time consuming. People are using words such as cruel and mean in relation to restrictions. It is hard to know how to respond so as not to inflame their fraustrations. There is a balance between letting people have their say and trying to prevent staff and clinical situations being identified.
Updates regards visiting mainly been published on social media. Wrongly updates have gone on her from matrons before midwives we even aware
The local breastfeeding group have been more active and have moved to using zoom to provide support for mums. I think this is a good idea in the circumstances but can't replace face to face support in the long term.
Conversely I spent less time on social media during the pandemic than I normally would so whilst I’ve been aware that digital support was going on I have really seen it and I haven’t been involved professionally online.
Many mums not presenting to assessment unit when they really should have done because they have followed advice found on FB groups. Poor outcomes for some babies
I have initiated, supervised & supported the expansion of volunteer led online services more than provided it directly myself - 5 geographical closed FB groups with trained volunteer admin teams 3 weekly zoom sessions led by BF counsellors supported by peer supporters Working collaboratively with 3rd sector colleagues is absolutely integral to providing a safe & effective BF support service
Virtual breastfeeding support
my local trust and the trist i have jad my placements in have created ask the midwife sessions to keep women up to date with current guidance on visitors, birth partners etc. I feel this has been a good way to ensure women are all told the same information, and stops hearsay.
N/A

Midwives and maternity services communicating with mothers and families via...

Our trust runs a monthly Facebook live where you can ask questions

N/a

Data protection and safeguading issues are common challenges

A sudden recognition by my employer of the value of what I had been doing for a long time in my spare time! Suddenly this was a role that warranted paid time, which felt like a kick in the guts to be honest. Everything I had done to date has been set up, maintained and learnt in my own time and I know it has been of immense value. During covid, we have expanded our services online and been able to involve more Midwives (those who cannot work clinically) - it has been of value, but I do not believe that it is truly seen for the potential it has - that is still a battle.

Virtual clinics, antenatal education, media/communication midwife post, attend anywhere breastfeeding service, all very positive and well received by midwives and service users alike

I have seen many trusts post upto date guidance on what is happening with regards to maternity care during covid. However, a lack of evidence to support what they have put in place and why which has been frustrating. They have also posted comments from other parents experiences of maternity care during covid which has been reassuring for some and upsetting for others who didn't receive good quality care to the same standard.

This blurs boundaries. Women can see your profiles and understand more information from you. I strongly advise against this. It can increase burnout.

I like the accessibility of online information and flexibility it brings

I signpost to RCOG guidelines and various helpful articles.

MVP group clamped down on 'negativity' in the face of perfectly reasonable requests. Local trust group just published trust propaganda. Non-NHS based groups were supporting huge numbers of very distressed people with nowhere else to go.

We haven't moved to online help. We've continued as we are

I feel privacy is a massive issue, and communication over digital platforms may not be the best thing for women who are very emotionally invested in breastfeeding and who may require support in a more personal way. Care isn't personal when it's through a phone or screen!

Our best start teams all have a Facebook group with the midwives and women, and the midwives and the small amount of women I spoke to about it all had really positive things to say

Useful to update women on service changes

I have signposted more through this time as there is such a lack of face to face services

Online virual breastfeeding support sessions by a local group - seems to have worked very well and praised.

I worry about false information being provided by group members and the groups not being "manned" 24hrs so therefore cannot counteract any concerns with evidence based information.

I feel that in some areas there's a reduction in midwifery input. This hasn't happened with me. I have had only one telephone consult with a lady isolating. All the rest - face to face. Proud of this. Have provided good care. Some women haven't been that fortunate

We have moved antenatal training online with very positive feedback

Videos and webinars are easy

Midwives and maternity services communicating with mothers and families via...

Very positive response from staff and patients

It is good for communicating information, but the comments need to be switched off. No one pays attention to 'rules' or 'guidance' when making comments. It is a litigious method of commu ication

The health board has been sharing positive birth stories shared by women- this has helped women to feel reassured about coming into the hospital during the pandemic.

Increased use of facebook hospital group to inform patients of ever changing updates - visiting restrictions, need to wear a mask, videos of what to expect

Ambivalent so far - time will tell

virtual antenatal classes, non routine appointments via virtual means, BF support via zoom and online tours of maternity units

Our breastfeeding teams have conducted ZOOM classes effectively However antenatal classes have suffered.

Saw some online videos for things such as breastfeeding information and general updates surrounding changes due to Covid. I thought they were very helpful for mother's and gave them an easier way to find out information quickly, generally information via videos on Facebook for mother's helped mother's get access to information easily

None

Huge amounts more interaction on social media. Lots more negativity and discussion around the restrictions - lots of comment threads berating midwives for asking women to adhere to restrictions in homes to allow safe postnatal visiting. Confusion around health visitors vs. Midwives and midwives criticised publicly when in fact was HV. Hospital restrictions criticised eg. sonography appts no partners. By far the largest type of coversation I've seen has been women discussing and diagnosing normal newborn behaviours as 'reflux' or 'CMPA'. This might be good in other situations except I have then seen many occasions of discussing and recommending medications and dosages, what to say at doctors appointments to "get what you want".

Much more engagement online meaning bulletin style messages get shared and spread around quicker. Takes some pressure off community midwives and advice lines who would normally take brunt of calls regarding changes -this is positive. Easier to get feedback and find out what is working and what isn't - also positive but not formalised.

Attend anywhere is great.

More digital resources, fb/insta used as a means of information dissemination and communication. More content for pregnant mums and families .

As a midwife of 7 years, I tried to offer support on a pregnancy group when women were asking for advice. This ended up in the woman not liking the advice given and prompted a suggested report to my employer and Nmc because I was accused of making the woman look stupid. This has been very off putting at supporting women online, caused undue stress which wasn't needed being pregnant myself, and resulted in me seeking support and advice from my trust SOM for transparency if a report came in from the nmc. I followed guidence and remained professional in my support given but this caused stress to myself. I will not offer online support again.

Less women were calling and attending the emergency triage unit which had a positive impact on the activity of the maternity unit. Having less visitors about the hospital and on the maternity has allowed mums and babies to bond easier. This has also benefitted the staff as they answer less door bells and phone calls and can complete tasks more efficiently.

lots of extra support online

We have seen huge numbers of our women turning to the Facebook group - it has been vital support for women during this time.

Many women have felt socially isolated during the pandemic.. Facebook pages and social media forums have helped to bridge the gap and provide support when they haven't had family or friends to be able to turn to.

n/a

We have a health board Facebook page monitored by the Consultant midwife supported by a communications team. Feedback from women and families is in the main positive. There is a breastfeeding Facebook forum however this was in place pre pandemic and is proving successful

Women seeking more online support during the pandemic and I feel midwives are responding to this is a positive way

I like the virtual parent education and meet the midwife sessions.

More people communicating on facebook posts etc mainly positive comments etc but not always - some mother's and families have expressed their disagreement with policies etc during this time such as visiting restrictions which can cause issues but overall more positive than negative comments have been seen by myself.

The ladies are getting a poor service according to my pregnant ladies, i often get the comment, ive leaned more from you in 1/2 hour than I have in my whole pregnancy.

I have pulled back as I am now a Midwife in my local area so hard to balance personal and professional. I'm also totally overwhelmed at work and feel I can't give any more at the moment.

I feel positive, it has been a quick and reliable way to share information with a large number of people. I have also seen some negative impacts, with free replies to posts it can sometimes create a platform for complaints. However, the majority of the time it works well.

It is good that midwives are accessible to women in a generation where social media is preferred to a phone call. Unfortunately this does mean there is a delay in some women accessing appropriate care, as they will message with a serious concern when the inbox is not manned (though it is widely publicised what times it is manned!). I also am concerned by the messages received by people who are seeking emotional support, when this would be best provided holistically by their community midwife. I feel in such cases, the Facebook midwife should inform the community midwife of their contact to follow up.

We have a Maternity page for our Trust that has been used to give info to women about changes to visiting, etc. It has been mainly used by women to slate the midwives and criticise us, which has upset a lot of midwives

Lots more positive birth stories which I hope has served to increase women's confidence about accessing midwifery care during this COVID year

I have seen an increase in students using FB for career development and learning opportunities. I have also seen more interaction from women who are unclear on COVID guidance etc etc and are seeking support.

Lots of well meaning women providing support but dangerous information on feeding etc

Online bf support - has worked well. Vcreate in nnu - better than nothing but you can't hug a screen

Its a positive step as it allows information to be accessed easily however there can be delays in seeing posts and answering questions etc

Service user collaborative meetings are better attended by women now they are virtual, more of a cross section seen.

Our services were set up prior to covid, however we have since seen a huge demand from other trusts seeking assistance and guidance on how to provide digital support safely. Women who have used our service have responded extremely positively and the has also been a huge positive impacts on Trusts involved. Women have been able to access information and support when there has been a lack of face to face support.

It is a step forward and I want to embrace it. I just am concerned about how to do it together as a team.

Just YouTube videos

The increased use of social media by women. Women relying more on community groups and increased activity on our Facebook groups as women seeking more support.

some midwives have not been socially distancing in the photographs

With all the changes happening so quickly out social media groups have been a great resource for women to get up to date information. Also I am apart of a Facebook admin who give pm advice to women (non-clinical) this has really helped women understand what is happening during the pandemic.

Another local trust has embraced using Facebook to communicate with the women and i have been impressed by their engagement.

In my research role we are trying to move group care online using Zoom (women-led discussions facilitated by 2 midwives, breakout rooms for 1-1 consultations, self-checking PB & urine etc instead if 1-1 telephone appointments). I've also seen midwives setting up WhatsApp groups for women due the same month then leaving the group (for peer support) - with consent, obviously! Our local BF support, safeguarding meetings, and GP/HV meetings have moved online and anecdotally are better attended than previously. Locally a few M W use AttendAnywhere for virtual 1-1 appts but most mw not confident or don't have the kit. At my Trust I cannot access Facebook or any external site from desktops and they don't have cameras or audio. I think there is great potential in terms of improving communication & cutting travel, and offering more flexibility to MW (working from home) but we must be wary of seeking and respecting women's preferences and not reducing screening (including supporting self-screening ie with BP/urine).

Those comms were seen as temporary during extremis. When all fave to face meetings can resume the concern is that they will not - women will be left with inadequate levels of care and midwives held responsible for mopping up issues via Facebook. It’s another nhs care scandal waiting to happen

Updates about covid guidelines are often posted on our trust maternity Facebook page, breastfeeding info is posted on there, free antenatal classes online are posted on there, 'meet the mideife' bios, photographs and resources. I worry 'meet the midwife' means people may try and 'friend' midwives online and this over step the ethical boundaries and put pressure on midwives to work outside usual hours and get involved in patients personally.

None

I have notice bf groups on fb being great sources of support for women who cant go to groups

My trust has a page that provides information to the public about current services and changes etc this is manned by an IT specialist who is not midwifery trained and has lead to some miss information. It has also lead to posts from the public 'naming and shaming ' midwives and care provided. We have a policy that is not very specific about social media use and I feel that leaves things open to individual interpretation which can prove tricky. I understand information sharing and social media being a good platform to reach lots of people easily however I feel that this will take away from women building relationships with their named midwives or contacting the correct service such as triage when concerns arise. I feel that as a midwife this would also increase pressure in my home life if I were to use social media and written text can be somewhat misinterpreted at times causing potential issues. If sending messages to patients there is the potential for them to become screenshots and used inappropriately. I prefer to speak to women directly rather than use text or social media

The trust have been able to see themes very clearly through our ‘ask a midwife’ service as multiple women ask the same questions

All positive if moderated professionally

We don’t do that.

Advice changed daily and so advice i gave was right one day ,wrong the next and then right the day after that. I became v worried. Also info given by dept staff was then contradicted by managers...vconfusing for everyone

More frequent use during covid which has been an excellent way of communication with women and families during times of frequent change

Unfortunately have been not been on placement so therefore have not been exposed to changes yet.

It’s something most mothers are comfortable doing. Can get breastfeeding support without leaving their home in covid situation taking unnecessary risk and stress out of it

offering antenatal classes online - a good alternative when face to face is cancelled

It hard to ensure evidence based guidance is provided and supported by some peer groups. They can be helpful but also provide incorrect advice

Increased content

Unsure as I shielded

None - have been delivery suite based.

During coronavirus many midwives help pregnant mother

Positive

My trust specifically made videos to update women on changes to services (I.e. visiting and support during inpatient stays), to provide tours of the intrapartum areas in the absence of physical tours, and information regarding services we provide. I think it's great to have these options for people unable to attend appointments for various reasons (lack of mobility/disability, mental health issues) but I don't think they should be the only option. Once the pandemic has passed, I believe we should provide both face to face services and information online to provide extra support. I don't think you can necessaily give all information in a forum that isn't as interactive/doesn't have any element of trust building between practitioner and pregnant person.

A positive has been that women have shared their birth stories with thanks and appreciation for the great care they have received- this improved morale for the midwives and provided other mums to be with some reassurance

women are less likely to visit triage if they experience a problem in pregnancy.

My trust hasn't used social media for actual midwifery care, apart from a small number of antenatal education sessions. It has mostly been changed to telephone / video appointments. I still feel like women benefit more from face to face contact than online contact.

We have signposted women to a support group offering bite size ante and post natal classes

It’s a great idea however, trust up for public verbal abuse and becomes frustrating when you can’t challenge the ‘keyboard warriers’

None

More reviews and stories from women

The sense of community on social media platforms between expectant mothers has been a lot more apparent during lockdown as women have been unable to attend face to face parentcraft/mother and baby classes etc and have been reaching out to each other a lot more online for advice and just general friendship.

Parent classes now videos in FB group. Definitely not the same as actual classes.

STMW, was pulled from practice, but have seen so much misinformation an conspiracy theories including related to maternity. Not helped by government incompetence and misdirection.

I feel like it's been useful to keep women up to date about their local services

Video calls. AMAZING!

Unsure

Social media is an excellent resource to communicate changes, etc to women. There are very few women who are not on social media so changes can be available instantly. Women use our page to ask question & seek guidance. We have a selected number of midwives from all areas within the hospital available to answer questions, ensuring the most up to date information is given.

I have concerns over midwives using personal Facebook profiles to provide advice as for many midwives this will be against their artist guidelines and however well intentioned could lead to implications to their employment and practice. Facemums was creating as a safe space for mother's to meet and to be moderated by midwives. This model has produced impressive results during lockdown with mothers reporting their gratitude for the service. But the experience for midwives can be stressful and I have concerns that without formal support such as the role I undertake or PMA support midwives can experience anxiety and stress in supporting women on social media.

Same as above really. Nastiness has got miuch worse. Poor information shared by women between each other, disprespect to mws and drs and other health service workers. Doulas and birth educators calling themsleves professionals and practitioners and constantly trying to sell their services as being better than what the NHS can offer but actually preying on women who are vulnerable. The same groups are attacking us as midwives. We even had a complaint from one doula about posting a graphic about the number of girl/boy babies born each month. We were posting it in response to mums loving this, but the doula says that it's not woke and we shouldn't be assigning gender at birth. Midwives have much better things to do than have to referree the behaviour of doulas on facebook, and yet that's what we're having to do. That and constantly try to address misinformation.

Women have needed reasurance on how to access the hopsital safely and that w are using PPE equipment. They have also felkt resaurred by other women success stories they have read on on our Maternity Facebook page.

I have seen breastfeeding classes online but women have stated they have found these mostly unhelpful as they do not receive feedback on what they are doing and are not being 'taught'

More video chats via WhatsApp , I think it's very useful

More use of twitter to spread messages. Women and partners making contact via social media to ask questions. More women making complaints via social media.

I don't have any burning feelings in regard to communication via social media….really it was inevitable.

In our trust we haven't had any changes-I feel we our behind the rest of the country!

Social media is a good accessible platform for women however a lot of the sites I see for women and midwives share a lot of conflicting advice and I see women asking for advice that requires immediate attention. I worry that social media is used instead of triage assessment and risk to women and babies is increased. I do appreciate however that it is a platform where women will ask questions that they may not feel comfortable asking in person so this is beneficial. The grey areas between advice and information and clinical episodes however can be difficult for women to understand and even one poor outcome causes by waiting for a response to social media post is too many. Equally Facebook may blur boundaries between professional and personal relationships.

not seen any in my area but good to see what others have been/are doing

Videos on BF support, safe sleeping and baby care. Very good videos, improves women's confidence at home, easy reference guide that can be trusted.

I havent seen any

I have seen mothers becoming more aggressive in their commenting on hospital guidance both against midwives implementing the guidance and each other

Unsure as not personally had to do

More people offering support however lines and boundaries I'm unaware of - I want to do this and I'm scared of disciplinary etc.

Not via social media but shielding midwives have been providing breastfeeding support via video link

Positive step, should have happened a long time before the pandemic, we have been ignoring the main way to reach women

I feel its easier for women to reach out to social media, particularly in the midst of a pandemic

Unfortunately this increased but women clearly were unsatisfied

.

Heavier and more widespread use of HB groups to provide information and support. The information is useful in dispelling myths etc but I have had to unfollow many of them (I was pregnant and gave birth during the pandemic) as I felt, and still feel, that there is alot of anger and vitriol on these sites. It's also difficult when you are seeing bad advice being given by others. I have also seen some quite unprofessional responses from the moderators of one HB group which only served to fuel the fire. Maybe it is hard for some to try and remain neutral when the pandemic has put so much pressure on a service? Then it feels almost personal when someone attacks it as they are talking about you and your work colleagues.

Trust set up new FB group which is very ineffective. Inactive most of the time and again for mothers only run by 2 midwives.

Trust Facebook page is regularly updates with guidance re visiting restrictions, face coverings, breastfeeding guidance, which has been really useful and fast, wide reach to women

I feel very concerned that that social media will replace face to face dissemination of information. A key skill for a midwife is to tailor both content and level of information to each individual to ensure it is relevant and understood.whilst I see the benefit of increased reach, a one size fits all approach will not tailor information for those who most need it

we have started an ask the midwife facebook page for women to be able to contact a midwife. This has been mainly questions around COVID however

There has been no changes in my area in scotland . No communication service for parentctaft , nothing provided for women

Not been in practice

I feel these were initially a fantastic way of giving the relevant information that was still very much needed during this pandemic, unfortunately they very quickly became a platform to criticise the health boards and Midwives etc which became very detrimental at an already stressful time

Haven't observed changes locally

FaceTime, Zoom and team appointments I feel they have both positive and negative sides. It depends how they are facilitated. Some appointment can be recorded so the women can listen back to information given. A negative can be it loses that one to one relationship

Access to baby feeding information via one to one online service.

antenatal classes online and online tours of facilities, although its not the same as being in person where you can ask questions and communicate more effectively. I feel everything online has been clearly presented and some questions have been answered in the comments section

I have seen lots of antenatal classes being uploaded by my local trust onto Facebook which I think is a really positive change as more women can access the classes and look back at the information

Seen Midwife's posting antenatal videos which have been great, though parents are unable to ask questions like they would in a room with the midwife

I feel social media is a two edged sword. The groups that I'm moderator of were already well established prior to Covid & are now Trust affiliated. I feel it is a safe space to communicate & we have strict rules of ensuring safety of all members I have found some of the other groups on Facebook less favourable - the all Wales antenatal page can be so informative & useful bit I don't feel it's moderated enough & sometimes can become quite negative & feel like a non-professional group. Our trust has a personal page also "bump talk" which is more closely moderated & posts have to be approved first by managers.

I feel they have been beneficial in communicating changes to policy/ apppintments

Increased usages of Facebook to ask questions

It has been necessary to find new ways of communicating with women and families at this time but it should never be a substitute for where women want to see someone physically outside of the pandemic. It is a vital choice as some women prefer to communicate virtual and this is great when it is for general information etc. It can't replace vital appointment when physical assessment is needed. It is vital it is carefully monitored to make sure that information is correct and that incorrect information is not shared as many mums groups already suffer from the sharing of incorrect info such as baby's movements etc. It can easily become a sound board for shaming each other etc feeding. However if managed correctly it can be an excellent addition resource to promote information about all aspects of pregnancy, birth and the postnatal period.

I feel digital skills have improved greatly and questioning archaic practices has been forced. Progress has been pushed forward which would have otherwise taken many years.

I've seen that a lot of women are better informed, and correcting other people in groups with evidence based information and links to genuine resources. It's been rather good to see that happening.

Infant feeding teams using web cams to give breastfeeding support to women from a distance, i feel these are convenient for women and midwives, as the women don't have to leave the house and midwives dont have to waste as much time commuting to different womens homes, however if women don't have anyone there to hold the camera, or if they don't speak great English it can be hard. Great for checking in with breastfeeding women postnatally, but probably not suitable for more complex cases or other postnatal care. Antenatal appointments over the phone do not work well in most situations in my opinion, some things have been missed that would not have been if the woman had been seen face to face, and some women feel less connected to maternity support.

People are starting to rely on peer support as NHS midwives are just distancing themselves from the community they are meant to be supporting

May have brought strangers closer together

Our women are using Facebook to seek support much more during Covid

It's become the new normal for women to reach out on Facebook in our trusts moderates group. I feel like it's been really nice to be able to help women connect more through this time. However, I feel terrified that I may get something wrong. Most of the time I would reply to a post by advising the woman to call labour ward or see her midwife. I've once given more support and I'm absolutely sure it was correct but I'm still terrified that I would have been perceived as unprofessional. I was super careful but I'm still worried.

Myself and my two colleagues who manage our trust Facebook maternity page had been inundated with questions re covid 19. I personally feel we have been able to offer clear information, answer FAQs and reassure women during the pandemic. This has also prompted families to share their positive experiences and reassure each other which is often more effective than professionals trying!

Not sure

The Antenatal Education support group on Facebook is an example of poor communication, security, privacy etc. Set up by consultant midwives who had little knowledge of Facebooks features. Women posting freely all over Wales. There is no screening and approving posts. Women are posting confidential matters and asking for advice. Quite often women will recieve incorrect guidance from other mothers. Midwives then feel obliged to correct it. Midwives have been contacted privately if women know that they are a midwife. There are no rules. The antenatal education stopped 2 months into COVID-19 lockdown. Complaints are not being addressed. Other pages run by organations have been more enlightening. And feel that women are getting the information needed during lockdown. Unfortunately there is little is the way of education as many midwives were busy supporting the busy units.

We have been able to connect virtually with mums.

Less stress for midwives using online video for booking

i feel there is a lot of offer and mothers get overwhelmed with everything they are offered

More antenatal education videos. This has been valuable in bridging the gap in the absence of face to face education and support. More midwives commenting from their personal accounts in response to questions on the Trust’s professional pages.

Lots of info is now being sent via email rather than printed- great change!

We have found the online support invaluable, now we are seeing other mothers providing the same information as we would. Our daily groups are run by breastfeeding support workers and overseen by midwives

Additional use of social media to promote changing guidelines around visiting hours. This has helped to feed information to a wider audience and provide consistent updates that are easy to access

Midwives presence on social media has become greater in order to ease women’s fears regarding COVID-19

Move towards digital appointments and documentation online - about time !

Weekly question and answer sessions on Facebook with midwives present for an hour or two. So much information sharing on these. Have also found Maternity Voices to be very good at sharing information online

Creating sense of community and regular updates have been important

More online resources

Our trust launched Ask A Midwife via the Maternity Services Facebook page and it was been well used and women reported finding it very useful

Think it’s a positive change, Midwifery keeping up with the times!!

Maternity group with feedback on birth experiences and breastfeeding support

More use of Facebook pages for maternity services

Women are desperate for support.

l have seen videos and q & a sessions both within my own trust and others which have been extremely useful and very effective. from showing women how to access a new unit entrance to how to settle a crying baby and physio sessions including pelvic floor exercises.

I have seen lots of Trust updates via FB. And local breastfeeding support meetings have moved online.

The trust I work for has been using facebook to update women on guidelines in regards to visiting the hospital, attending appointments alone and schedule of care. I feel it has been a positive change as it has allowed women to stay up to date with the changing guidelines.

I have seen more positive birth stories shared online which is lovely. But also women posting with pregnancy concerns that they should have directly called the assessment unit with rather than delaying and posting on the group.

The impact on mental health has led to women looking for other sources of information/support such as that of Facebook. Facebook as a support network for women has become more increasingly used. A lot of women have been sharing positive experiences around labour and birth online to help reassure other pregnant women, and also to thank the staff involved in their cares when in the unit as inpatients. I feel Facebook and the ability to share advice and support has been a more supportive for women and midwives during the pandemic

All wales antenatal education group has been brilliant for women to get in touch with each other and receive advice from midwives and hear birth stories at the same time

I've really liked seeing posts by women shared by the official HB page thanks midwives and students for the care they received as well as seeing photos of the babies at home with their families. It has been lovely to see and read about all the positive experiences women have had despite the pandemic crisis.

Not social media bit a telephone line with video link 24/7 has been really helpful and lessens the burden on the postnatal ward answering these calls

IBCLC facetime appointments - appear helpful.

Increases use of social media an internet to get information, advice, remote appointments. Definitely it is the way to go forward as much as practically possible

my trust has used their maternity Facebook page to announce corona virus updates. Often the midwives would learn of changes through facebook ahead of direct email communication. E.G the suspension of homebirths, the no partner on wards etc. In some circumstances it felt unprofessional to explain to women they would need to follow the fb page in order to know whether partners were allowed to attend scans etc as I was not always able to give them that information at the time of them asking, however, it has meant women would find out updates as they happen rather than having to wait 2 weeks until they next see their mw. I did notice that as trust decisions became more and more unpopular (no partners to scans for example) there seemed to develop a 'pack mentality' among the public responses and what had started off as a useful poof information where everyone was grateful for keeping them safe, it soon turned quite angry with what you might call keyboard warriors. Also of course midwives employed by the trust were not allowed to respond to any comments or give additional information

None in my trust. We have a helpline on the phone and via email that we use with our women

N/A

Virtual classes and q&a sessions advertised on Facebook

Our family assist group for women and families can be a general moaning group and is easily dominated by strong voices.

None as I haven't been on placement during the pandemic.

none

Feel good about speed of change and use of IT in midwifery context.

I have not seen any

I have been taken out of placement so have not had the opportunity to see how care has changed during the pandemic

our trust has a facebook page which people can follow and regularly post updates, which can promote discussion

Lots of positive birth stories, women empowering women , women providing g reassurance for other women. Praise for the staff. Support for feeding/new mothers.

At the beginning of the pandemic, the All Wales Antenatal Support group on Facebook was very popular and helpful in supporting women through new social distancing guidelines, support for attending hospitals and advice on service availability and there was a lot of involvement with senior midwifery staff at the beginning. However, groups such as these are now overwhelming ly more like a “Mums Net” page and there appears to be little information sharing, far less professional advice on offer and now have become more of a parent support group in which often false, discriminating and unnecessary information is being shared which seems to be removed from the initial purpose of the group. It is how positive to see women sharing their stories and supporting and praising the NHS

I thin social media is a great additional platform for support but should not prevent support in person either in groups or one to one

Updates to services being provided have been regularly posted on my local Trust Facebook page. It has given women a central hub to find information & as much as I think it has been brilliant getting information to women in this way, it has also sometimes created a ‘mob mentality’ when discussing unhappiness with current guidelines. It has also spread negativity, worrying women who are yet to have their baby.

.

Set up social media project using Facebook, Instagram and Twitter with a weekly theme. Supported by other services such as speech and language, school nursing, occupational therapy, cahms, perinatal mental health team.

More frequent posts from support groups for mothers.

More focus on online links to give to women as not seeing them face to face

Supporting mother's on a more virtual basis when questions arise

I have seen other accounts set up but feel the traffic on the site is maker lead which closes down discussion which is a shame.

Lots of mothers missing out on early in person support before accessing our group.

Lots more posts than normal

y

Q26 - Is there anything further you would like to add?

Is there anything further you would like to add?
The questions in this survey seem to mostly relate to NHS midwifery. I don't use social media to support women as part of my (part time and quite recent) NHS role but as part of my self employed role. I don't have my current NHS role on my FB profile. I have a private FB group for my own clients. I also support women in FB groups that have started / grown during Covid as a direct result of reductions to NHS services. Some of these came about from community groups I supported in person before Covid. FB groups can be really useful for peer support but there can also be a cacophony of 'advice' that is not evidence based. I offer information, rather than advice, and encourage people to seek formal support from their HCPs, peer supporters, charities, independent practitioners etc as appropriate. I do a lot of signposting online. I think this is important from a consistency perspective and also re indemnity.
No
I think social media is an opportunity for women (dare I use the word) to share experiences of pregnancy birth and parenting but the fear of being shame by keyboard warriors frightens many mothers and professionals
No
No thanks
No
No
No
This took longer than 5 minites
No
N/A
No
I am aware though and mindful that not everyone has access to the internet or chooses to use Facebook so it should be used ‘as well as’ not ‘Instead of’
No
Improved communication
Groups are only as good as the moderators. Some are awful. Some are great. NONE should be permitted to silence women's voices.
A really good medium for supporting mother, midwives and especially students
I think this research is great and needed! I’d love to build my social media usage to aid my practice
No

Is there anything further you would like to add?

Just a concern for women's views - some may feel intimidated by peers and there is already so much pressure on becoming parents that it may cause an increase in judgement and feeling right or wrong etc.

.

Regrettably, the most logical prediction is that the use of such media will result in a further tightening of resources elsewhere in the profession.

an app developed to aid women who have language barriers or virtual spaces where they can be given information in their native language

No

Midwives still need face to face contact frequently to build a good relationship and assessment of the woman and baby.

Iam not confident most but not all families use it and find it helpful

Effective use of digital tools requires a team of midwives to be competent not for the responsibility to fall on one or two people - that is when it become draining and doesn't have the same continuity benfits etc.

Midwives should use social media with caution. When information.ing women regarding social media groups women should also be aware of the majority of information provided by the public may be incorrect or non-evidence based. I would always recommend to women to contact a midwife for support

I think the younger Midwives will grasp the nettle and it could be a success only if adequate training is given.

I would love to provide this support. Sadly we are really short staffed so would have to pull staff out if fresh air :(

Social media should be ingrained in our work. I believe each trust should have a social media manager Midwife, and it should become a recognised post. Alongside this I feel there should be more clear guidelines and support around the country from specialist, non clinical, social media managers - so that we can use this free and far reaching tool to our advantage.

Good luck with your research

Although I am not confident myself, and don't enjoy social media, I can clearly see it's potential benefits for mothers. Mothers can be signposted to many useful resources by its use. However, personal contact is still much more effective for teaching mothers the practical skills of breastfeeding, if it can be achieved, eg in Mothers' groups or Specialist clinics.

My practice hasnt changed during the Coronavirus - just got busier

Is social media supposed to be 24/7 accessible? Do the official social groups work certain hours? Do the women expect an instant response? Are midwives protected by their employer when giving this service? I have so many questions!

I have my own personal account that I use for personal reasons only. Very limited in the amount of Facebook friends that I work with on this account and I am not part of any "work" related groups on this. I set up a separate account for work use, where I share work related information, respond to women and families with this account only. This has an illustration as a profile picture so I am not identifiable, trying to protect myself more than anything else. And making this easily transferable if I were to leave this role and for someone else to pick up.

poor social distancing seen in personal photographs on Facebook (not only on the Health Board sites)

There are different issues on different platforms.

No

Is there anything further you would like to add?

No

I just worry that professionals can forget boundaries exist on social media. Especially when they post to each other...forgetting patients may see their posts too

Overall I’d be happy if my profile was just Midwife. Not if my name was on it

No

No

No

I do think social media has some benefits, such as providing trust based information, sign posting people to affirming support groups such as birth without fear, or support groups for perinatal loss, having a place they can look up information they might forget. But I also think it can be problematic: unfortunately some pregnant people will rely on advice from social media rather than calling their trust to discuss concerns and this can result in potential delay in treatment that could have impacts on their health and that of their baby, people can give advice that may be guideline based at their trust but not at the one the woman is booked at, they may give them information based on their skills and experience without informing people to see if their trust has midwives/doctors with similar experience (I.e. if I wanted a vaginal breech birth, I would not birth at my current trust but I would at the trust I trained at because they have more experience in supporting it and therefore more skilled staff in that area), and I think even though we should all be giving evidence based information, some areas have a lack of good supporting evidence, trust guidelines vary widelt based on the same evidence available so there is such a variation in information thay can be provided and personal experiences/opinions will always influence how you provide that information which may be difficult to navigate for families taking advice online into their experiences.

Social media has its place to provide general advice and announcements regarding the service however I feel we must be careful that it doesn’t take the place of specific and personalised care

no

Good luck with your rsearch

No thank you

The pandemic has shown that a lot of what was thought to be best done in person can be done online just as or more efficiently. This does need to be assessed in maternity so we can provide the easiest and most relevant services that maintain highest levels of safety.

No

I was part of a baby group when I was pregnant, we were all due at the same time. There was a lot of people posting questions, is this normal etc. It would be nice to have a midwifes input on groups like that to keep everyone safe. Sometimes people won't ask their midwife about something because they don't want to bother them by ringing them up, but they might feel more comfortable asking in a support group.

The problem with social media comes if midwives answer questions, make comments using their personal social media account. We emphasise to midwives that they should not respond to messages, good or bad, using their personal log ins. Any bad comments are referred to our communications team to deal with.

Thank you and good luck with your PhD!

I am incredibly passionate about the benefits of Midwives engaging With mothers on Facebook and social media. But I feel this must be undertaken as professionals not on personal accounts as the line that blurs between professional and private has potential repercussions for the midwife’s professional career. There are professional boundaries to maintain in accordance with NHC and Trust guidelines. I fear bullying of well intentioned and brilliant midwives on social media puts off other midwives from engaging in social media and I hope that this research and others like it supports a more realistic assessment of the benefits of engaging in social media for our profession, whilst encouraging midwives to find a balance when engaging in social media to protect their work/life balance and well being. Sending all the very best wishes for your research

Is there anything further you would like to add?

Need a 'whole family' approach to social media. Dads have been making more contact than the mums during lockdown via social media. They are feeling so left out and powerless.

No, thank you

no

I would always prefer a woman to contact myself or an out of hours midwife directly with a concern rather than await social media support.

Yes social media and support this way is the way forward - however most of the NHS aren't acting fast enough in my opinion

Health board management in midwifery do not know this generation of women. They fail to understand the increased need for contract, high rates of anxiety and depression and the needs of women both as in patients and in community. I would say women today have very different expectations of care from ten to fifteen years ago and the service has not kept up with this.

.

No

Facebook can be great to support clients and increase their knowledge but midwives are scared to support women due to politics and repercussions.

More education is needed to empower women with skills to distinguish between evidenced based information and opinion.

No

My local trusts Facebook page is open for comment to anyone who is a member, frequently other patents are answering and providing non-evidence based advice that has the potential to cause harm, before a midwife has been able to respond to the post. Therefore, it would be helpful for commenting to be turned off on posts and only for trained professionals to answer.

No

Twitter is my favourite social media platform for connecting to other professionals and agencies- Facebook groups need to have Maternity staff as moderators - being part of closed midwifery fb groups has rendered me in trouble with my Trust as I stated that I had palpatations once returning from sick leave - actually due to menopause ! Not asked if I needed support - just that I was potentially putting the Trust in a bad light!!

Completely support Facebook group for mother's however feel that midwife's running them/providing advice/moderating is a recipe for disaster!

Social media is about immediate information. Some social media that is being used currently is being held back by ownership and a need to review and provide bilingual information meaning that the updates are not always current.

There are roles outside the system for mws apart from independent midwifery.

My trust have been very unsupportive when I have done anything online, my trust have become very resistant to me growing and developing within my unit in the last 4 years that I've been "public" with what I do. It has led to a growing divide between me and them, and has been one of many things that is adding to me leaving the trust. However, the women who have had support from me on my personal midwife Facebook page, allow me to provide continuity of care (when it's needed), and achieve a Sense of fulfilment from being a midwife That my job no longer gives. I've offered my skills to the trust during covid 19, as I'm also in the clinically vulnerable group so have not been patient facing for months, and they have refused every step of the way. I'm heartbroken that the advances that could be made online are almost seen as witchcraft despite out trust rushing to swap antenatal classes. Covid 19 could have yielded major change for our trust, but instead I feel it's clinging to the dark ages.

The question "How do you feel about" privacy, professionalism, etc didnt make sense in conjunction with the answers.

Is there anything further you would like to add?

I am interested to hear your results

Our lecturers have given us extremes of views on social media use, probably good to have the spread but it has been confusing. Most of my cohort are like me, very worried about what we post online to the point where we probably wouldn't. This would mean women miss out on that advice/connection. I think we need more training other than scaremongering. I think the NMC should have really clear cut advise about social media use and whether students/midwives should be using social media to connect and when it's appropriate. This could stop me worrying about getting it wrong.

Very rarely get negative comments, more so lately with updates on covid restrictions. Will always respond no matter how uncomfortable the questions and comments and sign post for complaints if necessary. HOM was happy to give myself and colleagues hours per month for our time dedicated. We closely abide by the NMC social media guidelines. We always ask families their consent before stories and pictures are shared.

No thank you. Great questionnaire. I will be very interested to see the results.

No

We always intended to start an online group, it has been overall a very positive experience

Not at present

No

How do you feel about the following concerns in relation to providing Facebook Group support? The phrasing of this question made it very difficult yo answer with the options available.

No

No

Nope

why is the nhs so far behind the rest of the country on using IT, and why has it taken a pandemic to make trusts use this medium more widely?

Universities should be embracing the digital age. Our only session focused solely on the risks to our PIN and potential fitness to practice hearings!

I think using social media to support women in pregnancy and beyond is a fantastic idea and I would definitely be on board to help.

It would be great if more roles could become available, I have seen some digital midwife roles advertised but it is not very widespread. I think it is a fantastic tool but attention should be paid to provide the right advice and to moderate the group appropriately

no

No

No

No

no

Is there anything further you would like to add?

Wording of multiple choice questions didn't make sense. Difficult to answer as agree/disagree did not apply

no thanks

.

Social media can be a very dangerous place for professionals and the public. Professional only or public only platforms I think work best as these seem to streamline information better. When these two groups are intermingled on social media platforms, messages can be misunderstood or misconstrued and this can be damaging to both parties involved

I am not sure I answered one of the sections as I felt as I was unsure as to the agree/disagree relation to the questions. But in regard to my concerns around using social media and retribution I have concerns around confidently of us as midwives(students) and the support we receive if posting online. Good luck with the research it is a very interesting topic.

.

Please also consider the Health Visitor role within breastfeeding, fundamental,y our role provides the most support for the longer term, rather than a 10 day involvement,

Great idea, hope it has some impact. Good luck

Alot of work midwives due isn't visible I think this is another example of a movement to a new media which health board are decades behind the population.

I found one of the questions above hard to understand, I've used "agree" for yes this is a concern and "disagree" for not a concern to me.

y

End of Report
